# Preclinical Evaluation of the Safety and Immunological Action of Allogeneic ADSC-Collagen Scaffolds in the Treatment of Chronic Ischemic Cardiomyopathy

**DOI:** 10.3390/pharmaceutics13081269

**Published:** 2021-08-17

**Authors:** Ascensión López-Díaz de Cerio, Iñigo Perez-Estenaga, Susana Inoges, Gloria Abizanda, Juan José Gavira, Eduardo Larequi, Enrique Andreu, Saray Rodriguez, Ana Gloria Gil, Verónica Crisostomo, Francisco Miguel Sanchez-Margallo, Javier Bermejo, Blanca Jauregui, Lluis Quintana, Francisco Fernández-Avilés, Beatriz Pelacho, Felipe Prósper

**Affiliations:** 1 Department of Cell Therapy and Hematology, Clínica Universidad de Navarra, 31008 Pamplona, Spain; aslopez@unav.es (A.L.-D.d.C.); sinoges@unav.es (S.I.); andreu@unav.es (E.A.); 2 Instituto de Investigación Sanitaria de Navarra (IdiSNA), 31008 Pamplona, Spain; gabizanda@unav.es (G.A.); jjgavira@unav.es (J.J.G.); 3 Center for Applied Medical Research (CIMA), Regenerative Medicine Department, 31008 Pamplona, Spain; iperez.27@alumni.unav.es (I.P.-E.); elardanaz@unav.es (E.L.); srodrigu@unav.es (S.R.); 4 Department of Cardiology, Clínica Universidad de Navarra, 31008 Pamplona, Spain; 5 Department of Pharmacology and Toxicology, University of Navarra, 31009 Pamplona, Spain; agil@unav.es; 6 Jesús Usón Minimally Invasive Surgery Centre (CCMIJU), Ctra. N-521, Km. 41.8, 10071 Cáceres, Spain; crisosto@ccmijesususon.com (V.C.); msanchez@ccmijesususon.com (F.M.S.-M.); 7 CIBERCV, Instituto de Salud Carlos III, 28026 Madrid, Spain; javier.bermejo@salud.madrid.org (J.B.); faviles@redcardiovascular.com (F.F.-A.); 8 Department of Cardiology, Hospital Gregorio Marañón and Instituto de Investigación Sanitaria Gregorio Marañón, 28007 Madrid, Spain; 9 Facultad de Medicina, Universidad Complutense de Madrid, 28040 Madrid, Spain; 10 Viscofan SA, 31192 Pamplona, Spain; JaureguiB@viscofan.com; 11 Naturin Viscofan GmbH, 69469 Weinheim, Germany; quintana@bio.viscofan.com

**Keywords:** myocardial infarction, adipose-derived mesenchymal stromal cells, collagen scaffold, safety, allogeneic

## Abstract

The use of allogeneic adipose-derived mesenchymal stromal cells (alloADSCs) represents an attractive approach for treating myocardial infarction (MI). Furthermore, adding a natural support improves alloADSCs engraftment and survival in heart tissues, leading to a greater therapeutic effect. We aimed to examine the safety and immunological reaction induced by epicardial implantation of a clinical-grade collagen scaffold (CS) seeded with alloADSCs for its future application in humans. Thus, cellularized scaffolds were myocardially or subcutaneously implanted in immunosuppressed rodent models. The toxicological parameters were not significantly altered, and tumor formation was not found over the short or long term. Furthermore, biodistribution analyses in the infarcted immunocompetent rats displayed cell engraftment in the myocardium but no migration to other organs. The immunogenicity of alloADSC-CS was also evaluated in a preclinical porcine model of chronic MI; no significant humoral or cellular alloreactive responses were found. Moreover, CS cellularized with human ADSCs cocultured with human allogeneic immune cells produced no alloreactive response. Interestingly, alloADSC-CS significantly inhibited lymphocyte responses, confirming its immunomodulatory action. Thus, alloADSC-CS is likely safe and does not elicit any alloreactive immunological response in the host. Moreover, it exerts an immunomodulatory action, which supports its translation to a clinical setting.

## 1. Introduction

Cardiovascular diseases (CVDs) are the leading cause of death worldwide. According to the most recent report from the European Society of Cardiology, CVDs remain the most common cause of death in Europe, accounting for 4 million deaths per year. The development of new drugs and better medical care improved the acute mortality rates and patients’ prognoses. However, the number of chronic patients is still increasing, exerting tremendous pressure on healthcare systems [1].

The therapeutic benefit of mesenchymal stromal cells (MSCs) has been extensively demonstrated in different experimental models of myocardial infarction (MI) [2]. Therefore, several clinical trials, including the recent MSC-HF, MESAMI, and ATHENA trials, have been performed in patients with chronic cardiomyopathies, confirming the safety and feasibility of autologous MSC transplantation [3,4,5]. Nevertheless, the benefits of such treatments have been modest at best, as poor engraftment and survival of the injected cells hinder their therapeutic potential [6]. Several approaches have been explored to improve cell retention, including the use of natural or synthetic matrices to facilitate cell support [7]. Among natural materials, collagen represents an optimal candidate owing to its remarkable biocompatibility and mechanical stability [8,9]. We have previously demonstrated long-term cardiac recovery after treatment with an adipose-derived MSC (ADSC) collagen scaffold (ADSC-CS) in a preclinical porcine model of chronic MI. Furthermore, the use of the collagen system results in a much more robust ADSC trophic action, positively affecting myocardial remodeling and revascularization [10].

In addition, the use of autologous cells from diseased patients may have reduced the therapeutic potential and quality of the treatment; thus, using allogeneic cells from healthy donors may be a more attractive option [11]. Furthermore, allogeneic cells can be prepared as an off-the-shelf product, significantly reducing the associated logistical burden and costs. In the case of MSC therapy, the use of an allogeneic source of cells can also be justified based on their immunoregulatory properties [12]. Several clinical trials have demonstrated that despite the allogeneic origin of mesenchymal cells, they are safe and beneficial to patients with chronic cardiomyopathies [13].

In this study, we developed a clinical-grade system using a bovine CS seeded with ADSC and examined its safety and immunoregulatory properties for clinical application. We demonstrated that the transplantation of allogeneic ADSC-CS in a porcine model of chronic MI is safe and does not induce an alloreactive response. Additionally, its immunomodulatory potential has been shown in vitro with a human ADSC-CS.

## 2. Materials and Methods

### 2.1. Characterization of ADSCs and Production of Cellularized Collagen Scaffolds

Rat and pig ADSCs were isolated and characterized as previously described (Figure S1) [14]. Human ADSCs at passage 3 were provided by 3P-Biopharmaceuticals (Noain, Spain) after extensive quality characterization. A non-cross-linked collagen type I scaffold of bovine origin (20-µm thickness) was created by Naturin-Viscofan SA company (Weinheim, Germany). The chemical and mechanical features of this scaffold have previously been reported by Araña et al. [15]. ADSCs were seeded at a density of 10^5^ cells/cm^2^ onto the scaffolds and cultured for 24 h before in vivo implantation. The 1 × 1-cm^2^ CS constructs were used for the rodent experiments, and the 10 × 10-cm^2^ CS constructs were used for minipig experiments.

### 2.2. Rat and Minipig MI Models

All the in vivo experiments were performed in accordance with the “Principles of Laboratory Animal Care” formulated by the National Society for Medical Research and the “Guide for the Care and Use of Laboratory Animals” prepared by the Institute of Laboratory Animal Resources, Commission on Life Science, National Research Council, and published by the National Academy Press, revised 1996. All the animal procedures were approved by the Institutional Committee on Care and Use of Laboratory Animals at the University of Navarra (Project codes: 083-14 (approval date: 06/2014); 144-14 (approval date: 11/2014) and 095-16 (approval date: 09/2016)). MI was induced in rats and pigs by permanent ligation of the left coronary artery as previously described [16,17]. Animals were implanted with ADSC-CS, as previously reported [10].

### 2.3. ADSC-CS Safety Assessment in Rodent Models

The putative toxicity elicited by the implantation of human ADSC-CS was assessed as per the Good Laboratory Practices (GLP) standards in an infarcted immunosuppressed rat model. Therefore, ADSC-CS was prepared with 5 × 10^5^ human ADSCs. A total of 128 adult immunosuppressed Rowett RH-Foxn1rnu rats (Harlan, Barcelona, Spain) (10–14-weeks old), with an equal number of males and females, were categorized into 64 infarcted and 64 non-infarcted in the study. Half of the infarcted animals were implanted with ADSC-CS (MI-ADSC-CS group), and the other half was implanted with only CS (MI-CS group). The non-infarcted animals were subjected to surgery; half of them were implanted with ADSC-CS (Sham-ADSC-CS group) and the other half was not implanted (Sham group). In each treatment group, animals were categorized into four subgroups depending on the analyses and/or timepoint of euthanasia: the acute toxicity (euthanized on day 2 post-implantation), subacute toxicity (euthanized on day 10 post-implantation), subchronic toxicity (analyzed on day 28 post-implantation without euthanasia), and chronic toxicity (euthanized on day 90 post-implantation) groups. Throughout the study, mortality, general symptomatology, weight, and food consumption of the animals were analyzed daily. Moreover, blood and urine samples were collected at all timepoints for biochemical and hematologic analyses. Moreover, macroscopic evaluation of the necropsies as well as organ weight and histopathologic analyses were performed.

Additionally, a tumorigenicity study was performed using 8–10-week-old adult Rag2^−/−^gc^−/−^ immunosuppressed mice (Jackson laboratory, Bar Harbor, ME, USA). ADSC-CS prepared with 5 × 10^5^ human ADSCs were subcutaneously implanted into the animals, and the animals, 5 males and 5 females per timepoint, were maintained for 3 and 8 months. Finally, the animals were euthanized, and the skin and muscle samples from the implantation region were biopsied and processed for further anatomopathological analysis by a qualified GLP laboratory (University of Zaragoza).

Finally, a biodistribution study was performed to determine the fate of the implanted cells. Rat GFP^+^-ADSC-CS (5 × 10^5^ cells/scaffold) constructs were epicardially implanted in 4 male and 4 female 10–12-week-old chronically infarcted immunocompetent Sprague Dawley rats. After euthanization at 7- and 30-days post-implantation, hearts were histochemically evaluated for the presence of GFP^+^ cells. An anti-GFP polyclonal antibody (diluted 1:500 in TBS; Invitrogen, Waltham, MA, USA) was used as the primary antibody and the EnVision™-HRP conjugated system (Dako, Santa Clara, CA, USA) as the secondary reagent to amplify the signal. GFP^+^ ADSCs were quantified in four serial sections of heart, spleen, lung, liver, kidney, ovary, and testicle organs.

### 2.4. Implantation of alloADSC-CS in a Preclinical Porcine Model of MI

Chronically infarcted Gottingen pigs (60–80 kg, male and female) were treated 1 month after MI induction with 50 × 10^6^ allogeneic pig ADSCs previously seeded onto a 10 × 10 cm^2^ CS. Another group of pigs was intramyocardially injected with the same number of cells or DMEM media (control group). Blood samples were obtained at baseline and 15, 30, and 90 days after implantation. Serum IgG/IgM and globulin/albumin protein levels were analyzed, and the NK cells, monocytes, B and T cells, as well as T-cell differentiation and activation were examined using cytometric tests. In addition, hearts were extracted on days 7 and 90 post-implantation for the histological analysis of the scaffold’s biocompatibility. Macrophage and lymphocyte infiltration was quantified in 5–8 heart sections obtained from the implantation site after immunohistochemical staining for CD3^+^ and CD68^+^ cells, respectively. Cell detection was performed using an anti-CD3 antibody (Abcam, Cambridge, UK) and an anti-pig macrophage antibody (BIO-RAD, Hercules, CA, USA) diluted 1:50 and 1:500 in TBS and 10% BSA, respectively. An EnVision^TM^-HRP conjugated system (Dako) was used as the secondary reagent.

### 2.5. In Vitro Coculture of alloADSC-CS and PBMCs

Human ADSCs at passage 3–4 (obtained from 3P-Biopharmaceuticals) were preseeded onto CS and cocultured with peripheral blood mononuclear cells (PBMCs) previously isolated from different donors. After 96 h of coculturing, the lymphocytes were obtained, and a flow cytometry analysis was performed to determine the proliferation, activation state, and intracellular cytokine production of CD4^+^ and CD8^+^ cells. Additionally, PBMCs were activated by adding PHA (1 µg/mL), IL-2 (300 IU/mL), or PMA/Ionomycin (0.5 µg/mL and 1 µg/mL, respectively) to the culture media to stimulate proliferation and activation of lymphocytes as well as cytokine production. Lastly, the lymphocytes cultured alone onto CS were utilized as control.

## 3. Results

### 3.1. Safety Assessment of ADSC-CS

The primary goal of this study was to examine the safety and immunological reaction induced by transplantation of a GMP-grade alloADSC-CS into the heart for its clinical use in patients with MI.

Three different rodent models were used to test the potential tumorigenicity and toxicity of ADSC-CS and analyze the fate of the ADSCs throughout the body after ADSC-CS transplantation into the heart. Experimental design and assessment was performed in accordance with the official guidelines of the Agency of Medicines and Medical Devices (AEMPS).

**Figure 1:**
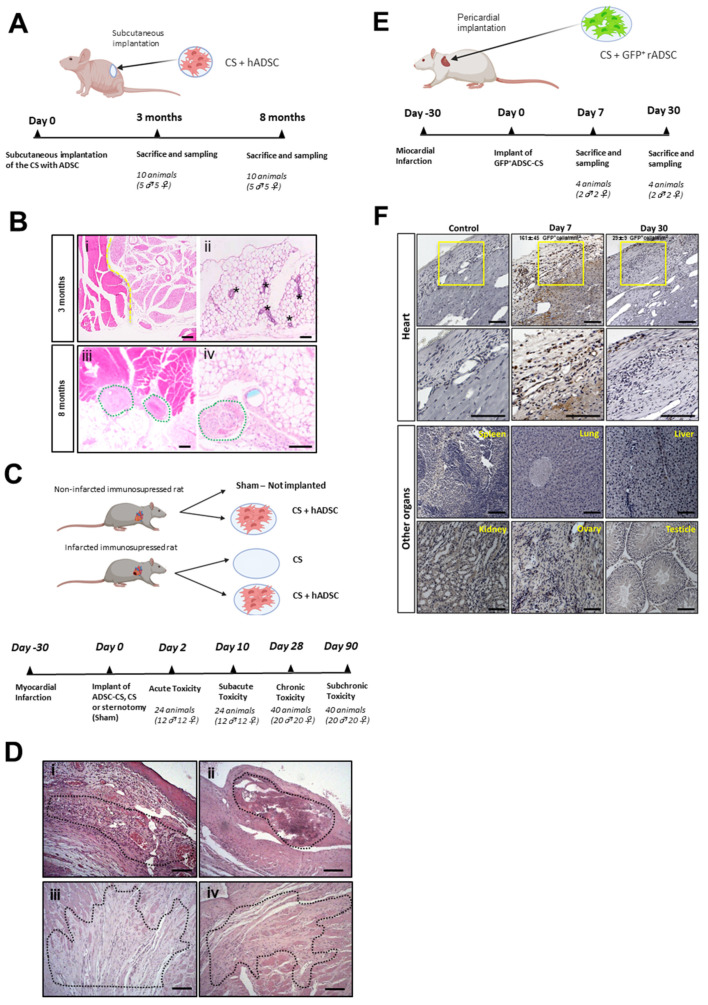
Safety analysis of ADSC-CS in rodent models. (**A**) Tumorigenicity study of the immunosuppressed mice subcutaneously implanted with human ADSC-CS. (**B**) Anatomopathological analysis of the mouse muscle tissues 3- and 8-months post-implantation. The representative images of H&E-stained muscle tissue sections exhibit (i) healthy skeletal muscle (left side) and brown adipose (right side) tissues, (ii) healthy white adipose tissue with mammary gland ducts (indicated by asterisks), (iii) suture material with moderate inflammation (green circles), and (iv) suture material in the implantation zone with a low-grade inflammatory response (indicated by green circles). Pictures scale bars: 200 µm (i,iii) and 100 µm (ii,iv). (**C**) The experimental design for the toxicological study in an immunosuppressed chronic MI rat model implanted with or without human ADSC-CS and CS. (**D**) The anatomopathological assessment of heart tissue sections. The representative images of H&E-stained heart ventricle show that (i) the MI-CS group with remains of matrix and myocardial necrosis (Grade 3) at the acute stage (day 2 post-implantation), (ii) the MI-CS group with mineralization (Grade 2), (iii) the MI-ADSC-CS group with fibrosis (Grade 3), and (iv) the MI-CS group with fibrosis (Grade 2) at the chronic stage (90 days post-implantation). Pictures scale bars: 100 µm. (**E**) The biodistribution analyses of ADSC after the transplantation of GFP^+^-ADSC-CS in a chronic rat model of MI. (**F**) The immunohistochemical detection of GFP^+^ cells (brown) at 7- and 30-days post-implantation in the heart, spleen, lung, liver, kidney, and male or female reproductive organs. Pictures scale bars: 100 µm.

First, the tumorigenicity of the clinical-grade human ADSC-CS was assessed by subcutaneous implantation of CS cellularized with 5 *×* 10^5^ human ADSCs in male and female immunosuppressed mice (Figure 1A). Tumor formation was not detected in animals sacrificed at 3- and 8-months post-implantation. Additionally, no ectopic tissue formation (bone, cartilage, or fat) derived from ADSCs was found at those timepoints (Figure 1B).

Next, for future clinical use, we examined acute, subacute, subchronic, and chronic toxicity associated with the implantation of the cellularized scaffold. MI was induced in male and female immunosuppressed rats, and human ADSC-CS (MI-ADSC-CS group) or CS (MI-CS group) were epicardially transplanted 1 month later. Two groups of healthy rats were implanted with the cellularized scaffold (Sham-ADSC-CS group) or left untreated (Sham group) (Figure 1C). The Irwin test was performed in all animals to analyze their general symptomatology, showing overall normal values (Tables S1–S4). No significant changes in food intake and weight were detected during the study period (Figure S2). No acute (2 days post-treatment) or subacute (10 days post-treatment) mortality was found in animals treated with CS or ADSC-CS. By 90 days post-treatment, 7 out of the 80 animals (2 to 3 rats/treated group) died, most likely owing to surgery or infarct induction. Blood, serum, and urine analysis showed no significant changes associated with CS implantation (Tables S5–S10) in comparison with the sham animals. After the animals were sacrificed, different organs were harvested, examined, and weighed. No significant changes were observed in the organs; only the infarcted hearts showed increased weight compared with the sham animals, likely owing to heart remodeling after ischemia (Tables S11 and S12).

The histological analysis of the hearts revealed ischemia-associated changes (data not shown). On days 2 and 10 post-implantation, remnants of CS were detected in the ventricle (pericardium) in the three groups implanted with CS or cellularized CS. Minimal pericardial inflammation was observed in the non-infarcted animals implanted with ADSC-CS, confirming the biocompatibility of the scaffold. Furthermore, infarcted hearts implanted with the scaffold did not contain any remnants of CS in the long term (90 days post-treatment). Meanwhile, only low-to-moderate myocardial mineralization was detected with the sporadic presence of pigmented macrophages and multinucleated giant cells, indicating a weak inflammatory reaction in response to the implantation of alloADSC-CS over the long term (Figure 1D, Table 1).

Finally, GFP^+^-rADSC cells were detected immunohistochemically in the myocardium 1-week post-implantation at 161 ± 45 GFP^+^ cells/mm^2^ in the implantation zone and 1 month later at 29 ± 9 GFP^+^ cells/mm^2^ in the same area (Figure 1E,F). Importantly, GFP^+^ cells were not detected in the spleen, liver, kidneys, lungs, or reproductive organs in any of the animals sacrificed after 1 and 4 weeks (Figure 1F), confirming the engraftment of the ADSCs into the heart without migration to other organs.

### 3.2. Inflammatory and Immunomodulatory Action of Allogeneic Pig ADSC-CS

**Figure 2:**
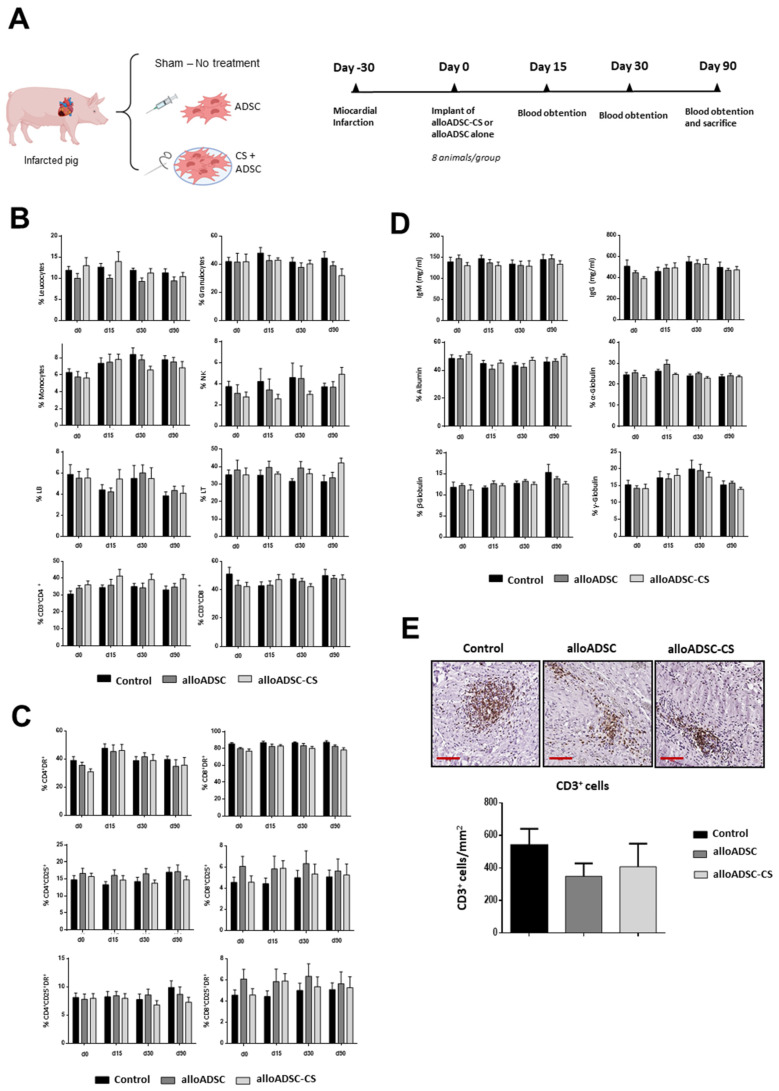
Immunological analysis in a preclinical porcine model of MI epicardially implanted with pig alloADSC-CS. (**A**) Experimental design. Adult pigs were implanted with only pig alloADSCs or alloADSCs seeded onto CS 1-month post-infarction. A third group was injected with a cell culture medium (Control). (**B**) Flow cytometric analyses of the blood leukocytes, granulocytes, monocytes, NK cells, and CD4^+^ and CD8^+^ lymphocytes (% of cells) before and 15, 30, and 90 days after implantation of ADSC-CS. (**C**) The analysis of the CD25^+^ or DR^+^-activated CD4^+^ or CD8^+^ lymphocyte subpopulations (% of cells) or both. (**D**) The measurement of IgG/IgM (mg/mL) and albumin/globulin (%) in peripheral blood serum. (**E**) The immunohistochemical detection and quantification of CD3^+^ cells (brown cells) in infarcted hearts 90 days post-implantation. Scale bars: 100 µm. Eight animals per experimental group were included in the study. The data are represented as the mean ± SEM. Statistical differences were not found among groups at the different timepoints (One-way ANOVA and Sidak multiple comparisons).

Next, we investigated the inflammatory and immunological effects of the implantation of a CS cellularized with allogeneic ADSCs. We compared the effect of the transplantation of 50 million allogeneic pig ADSCs or the same number of cells seeded onto CS in a porcine model of chronic MI. A sham-operated group was used as an additional control. Peripheral blood was obtained at different timepoints after transplant and were analyzed by flow cytometry (Figure 2A). No significant differences in the percentage of monocytes, granulocytes, NK cells, or leukocytes were observed among the groups at different timepoints (Figure 2B). Additionally, no significant lymphocyte activation was detected in different subpopulations among the three groups (Figure 2C). Furthermore, no significant differences were found in the levels of serum IgG and IgM or globulin/albumin proteins, confirming the absence of an inflammatory reaction against the allogeneic cellularized scaffold (Figure 2D). Consistent with the studies performed in rats, no significant changes in renal or liver function tests or other biochemistry tests were found (Table S13). Finally, local inflammation associated with transplantation of CS or ADSC-CS was assessed 90 days after implantation. No significant increase in the CD3^+^ lymphocytes was observed in the ADSC-treated hearts compared with the controls (Figure 2E), and almost no macrophages were detected (data not shown).

### 3.3. In Vitro Assessment of Human ADSC-CS Immunomodulatory Action

**Figure 3:**
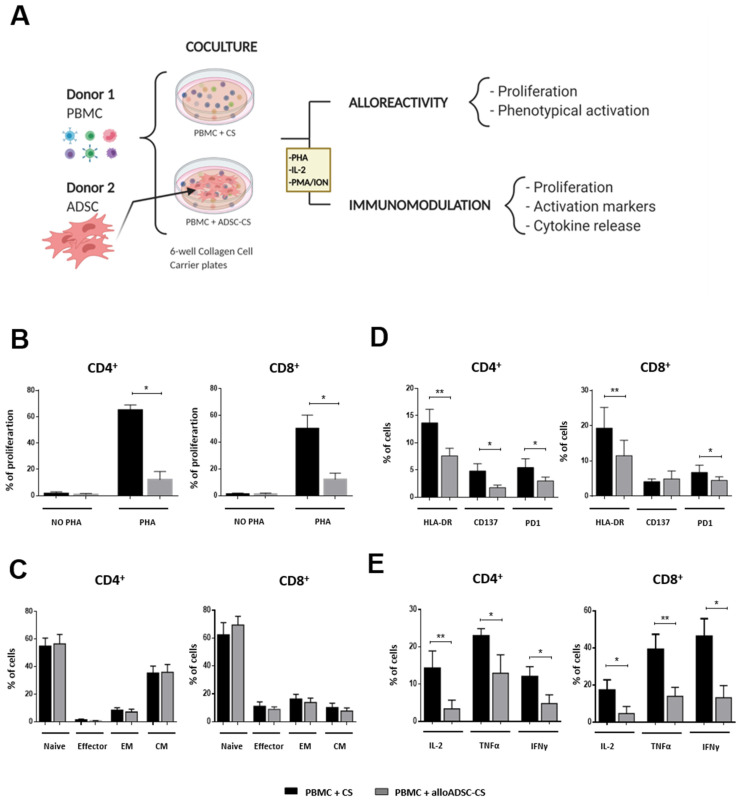
In vitro assessment of alloADSC-CS alloreactivity and immunomodulation. (**A**) Experimental study design. Human PBMCs were cocultured with only CS or CS previously seeded with human allogeneic ADSCs for 96 h. Subsequently, lymphocyte proliferation rate, activation, and differentiation were determined by flow cytometry. (**B**) The percentage of proliferating CFSE-labeled lymphocytes (stimulated with 1 µg/mL PHA or unstimulated) in the presence of alloADSC-CS or CS. (**C**) The percentage of lymphocyte phenotypical differentiation into naive, effector, effector memory (EM), or central memory (CM) cells in the total number of CD4^+^ or CD8^+^ cells after coculturing with alloADSC-CS. (**D**) The quantification of HLA-DR, CD137, and PD1 activation markers (% of cells) in the CD4^+^ and CD8^+^ subpopulations after incubation with 300 IU/mL IL-2 and coculturing with alloADSC-CS or CS. (**E**) The production of proinflammatory cytokines, IL-2, TNFα, and IFNγ by CD4^+^ or CD8^+^ lymphocytes after activation with 0.5 µg/mL PMA and 1 µg/mL ionomycin and coculturing with alloADSC-CS or CS. The data were obtained from five or six independent experiments. The results are expressed as mean ± SEM. * *p* < 0.05; ** *p* < 0.01 (ratio paired *t*-test).

Based on the safety profile of the allogeneic CS in the preclinical model, we analyzed the potential immunological response to alloADSC-CS in humans in further detail by performing additional studies in vitro. Human alloADSC-CS was cocultured with the PBMCs isolated from different human donors to determine its impact on activation and proliferation of lymphocytes as well as immune response (Figure 3A). Culture of allogeneic ADSC-CS did not induce alloreactivity in CD4^+^ or CD8^+^ lymphocytes. Conversely, coculturing with allogeneic ADSC-CS significantly inhibited the proliferation of PHA-pre-stimulated lymphocytes, demonstrating the immunomodulatory properties of the cellularized scaffold despite its allogeneic origin (Figure 3B). Additionally, coculturing allogeneic ADSC-CS with PB lymphocytes for 96 h had no impact on the percentage of the different subpopulations of T cells (naive, effector, effector memory, or T-central memory) (Figure 3C). Conversely, the expression of the activation markers HLA-DR and programmed death-1 was significantly decreased in both IL-2 pre-stimulated CD4^+^ and CD8^+^ cells when cocultured with the allogeneic ADSC-CS. Similarly, the expression of the CD137 activation marker was significantly decreased in the CD4^+^ lymphocytes, suggesting a potent immunomodulatory action of the ADSC on lymphocyte populations (Figure 3D).

Finally, allogeneic ADSC-CS induced a significant decrease in the production of proinflammatory cytokines IL-2, TNFα, and IFNγ both in CD4^+^ and CD8^+^ lymphocytes after activation with PMA/ionomycin compared with CS (Figure 3E). These results indicate the potential of the allogeneic ADSC-CS to modulate inflammatory reaction.

## 4. Discussion

The therapeutic benefit of MSCs has been demonstrated in the treatment of ischemic cardiomyopathies [2]; however, the limited engraftment and poor survival of the MSCs injected into an ischemic heart hindered the efficacy of the treatment. The use of scaffolds and polymeric supports to provide anchorage to the cells, a straightforward approach to circumvent this limitation, has already been tested in patients with CVDs [7]. Indeed, we successfully demonstrated a robust therapeutic benefit of ADSCs when transplanted with a CS in a preclinical porcine model of MI compared with cells without CS. The functional improvement in cardiac function and myocardial remodeling after ADSC-CS transplantation was associated with increased cell engraftment [10]. The translation of these results to a clinical setting required us to create our cardiac scaffold following all the GMP regulatory and quality requirements and test its safety as a therapeutic product. No relevant acute, subacute, subchronic, or chronic toxicity responses were detected after the implantation of the scaffold into the heart under GLP conditions. As expected, tumor formation or ADSC-derived ectopic tissue formation was observed even over the long term.

Additionally, we confirmed that the cells engrafted in the heart did not migrate to any other organ. As previously reported [10], we observed a robust retention of the cells using the scaffold system, with the cells still being detected 1 month after their implantation. All these safety results are consistent with the findings of previous analyses of the biocompatibility of the CS, in which a significant tolerance to the scaffold was observed in rodent and minipig infarct models [15]. Our data are also in agreement with similar analyses of this CS in rat and porcine models of urethral stricture, showing optimal integration of the urothelium–matrix constructs into host tissues with no toxicity, adverse secondary effects, or inflammatory responses [18,19].

Once the safety of the scaffold was confirmed and because we were willing to use allogeneic cells that could be advantageous in clinical applications, we analyzed the inflammatory and immune response toward our alloADSC-CS in a preclinical chronically infarcted porcine model. Although MSC immunoprivilege has been extensively observed, some preclinical studies have reported some degree of immune activation/recognition and response by the host following allogeneic MSC infusion or implantation [20,21,22]. Here, we found no relevant immunological abnormalities in our immunocompetent pig MI model after pig alloADSC-CS transplantation. In addition, no significant systemic changes were found over time, and no long-term inflammatory reaction in the implanted area of the myocardium was noted. In previous studies [10,15], we have demonstrated CS degradation within 3–4 weeks as well as a reduction in the number of engrafted cells over time. Together with the observed collagen biocompatibility and ADSC immunotolerance, these data can explain the lack of chronic inflammatory response toward alloADSC-CS. Moreover, the immunomodulatory action of ADSC may regulate any inflammatory reaction. Furthermore, no exacerbated acute inflammation was found at the heart implantation site (data not shown). This result was expected because in previous studies, macrophage infiltration was detected to a moderate extent at 2 and 10 days after CS implantation [15], a phenomenon which is in fact milder than that noted with several other natural and synthetic scaffolds [23]. Notably, a prominent anti-inflammatory M2-macrophage phenotype was induced by CS, likely beneficial for cardiac recovery after the ischemic event [23].

The results of our preclinical study with alloADSC-CS corroborate with the results observed in the clinical trials testing allogeneic MSCs in patients with ischemic LV dysfunction. In phase I of the PROCHYMAL trial, a good safety profile was found in the treated patients [24]. Similarly, the POSEIDON trial, a phase I–II early-stage study, showed no major alloreactivity against the exogenous BM-MSC, with low alloimmune reactions in patients (3.7%). Additionally, the incidence of adverse events was extremely low, and improved cardiac function and reduced infarct size were documented [25]. Similarly, in a more recent dose-comparison study with allogeneic MSC in patients with ischemic cardiomyopathy (the TRIDENT study), no serious adverse events were found, and the highest dose was reported to have a beneficial effect [26]. Moreover, a recent trial involving patients with anthracycline-induced cardiomyopathy heart failure (CCTRN SENECA Trial) also revealed an acceptable safety profile of MSCs [27].

Finally, we performed an in vitro coculture of the cellularized CS with allogeneic PBMCs to better understand the immunological response elicited by the alloADSC-CS in the human context. Lymphocytes play a key role in allograft rejection through the detection of allogeneic surface antigens [28]. Thus, we assessed the putative immune reaction elicited by lymphocytes toward alloADSC-CS. Because lymphocyte allorecognition of exogenous cells requires costimulatory molecules presented by activated macrophages, dendritic cells, or other antigen-presenting cells [29], we established a coculture of ADSC-CS with PBMCs containing the entire immune cell population. Our overall results did not show any lymphocytic immune reaction toward ADSC-CS. Moreover, we demonstrated that alloADSC-CS could suppress the proliferative lymphocyte response after mitogenic stimulation and the release of proinflammatory cytokines by lymphocytes after PMA/ionomycin stimulation in vitro. Additionally, the lymphocyte activation markers DR, CD137, and PD1 were reduced when activated T cells were cocultured with alloADSC-CS. All these data are consistent with the results of previous studies in which lymphocyte proliferation and activation are regulated by MSC cultures derived from different tissue sources [30,31]. Thus, all the immunological effects exerted by our cellularized CS can be beneficial in the ischemic environment of an infarcted heart, in which an excessive immune response leads to a deleterious remodeling process and a dysfunctional myocardium.

Thus, the safety of ADSC-CS demonstrated in rodent models, the immunoprivilege and immunomodulatory action of ADSC-CS attained in the allogeneic porcine infarct model, and the data from the in vitro human experiments in this study, with the previously demonstrated therapeutic benefit of ADSC-CS, support the clinical testing of alloADSC-CS in an ongoing phase 1 clinical trial (Nº EudraCT:2017-004503-49) with patients with chronic cardiomyopathy. Hopefully, data obtained from this clinical trial will validate the safe use of our therapeutic bioengineered cell-product in humans as well as confirm its potential as an effective therapeutic agent for the treatment of patients with chronic ischemic cardiomyopathy.

## Figures and Tables

**Table 1:** Microscopic findings in the hearts of each group and sex. The incidence (number of rats) and average degree of severity of main findings (in brackets) are shown. The degree of severity was assessed according to the following scale: Grade 1 (minimum); Grade 2 (mild); Grade 3 (moderate); and Grade 4 (high).

Time-Point	Findings	MI-ADSC-CS		MI-CS		Sham-ADSC-CS		Sham	
**Day 2**	**Sex** **(N° of animals)**	**Males** **(3)**	**Females (3)**	**Males** **(3)**	**Females (3)**	**Males** **(3)**	**Females (3)**	**Males** **(3)**	**Females (3)**
	Remains of matrix	3	3	3	3	3	2	-	-
	Inflammation, acute, pericardium	3 (2.3)	3 (1.3)	3 (1.3)	3 (1.3)	3 (1.3)	1 (2.0)	-	-
	Myocardial necrosis	3 (3.0)	3 (3.0)	3 (3.0)	3 (2.7)	-	-	-	-
	Macrophage aggregates	3 (3.0)	3 (1.7)	3 (2.3)	3 (2.0)	3 (2.0)	3 (1.3)	-	-
	Pigmented macrophages	-	1 (1.0)	3 (1.0)	3 (1.3)	-	-	-	-
	Epicardial fibrosis	3 (1.3)	3 (1.7)	3 (1.7)	3 (1.0)	2 (1.5)	2 (2.0)	-	-
	Multinucleated giant cells	-	-	-	-	-	-	-	-
**Day 10**	**Sex** **(N° of animals)**	**Males** **(3)**	**Females (3)**	**Males** **(3)**	**Females (3)**	**Males** **(3)**	**Females (3)**	**Males** **(3)**	**Females (3)**
	Remains of matrix	3	3	3	3	3	2	-	-
	Inflammation, acute, pericardium	-	-	-	-	-	-	-	-
	Myocardial necrosis	3 (3.0)	3 (3.0)	3 (3.0)	3 (2.7)	-	-	-	-
	Macrophage aggregates	3 (3.0)	3 (1.7)	3 (2.3)	3 (2.0)	3 (2.0)	3 (1.3)	-	-
	Pigmented macrophages	-	1 (1.0)	3 (1.0)	3 (1.3)	-	-	-	-
	Epicardial fibrosis	3 (1.3)	3 (1.7)	3 (1.7)	3 (1.0)	2 (1.5)	2 (2.0)	-	-
	Multinucleated giant cells	-	-	-	-	-	-	-	-
**Day 90**	**Sex** **(N° of animals)**	**Males** **(5)**	**Females (5)**	**Males** **(5)**	**Females (5)**	**Males (5)**	**Females (5)**	**Males** **(5)**	**Females (5)**
	Remains of matrix	0	0	1	0	0	1	-	-
	Inflammation, acute, pericardium	-	-	-	-	-	-	-	-
	Myocardial necrosis	-	-	-	-	-	1 (2.0)	-	-
	Macrophage aggregates	-	-	-	-	-	-	-	-
	Pigmented macrophages	4 (1.5)	4 (1.0)	2 (1.0)	1 (1.0)	-	3 (1.0)	--	-
	Epicardial fibrosis	5 (2.4)	4 (1.8)	-	-	2 (2.5)	5 (2.2)	-	-
	Multinucleated giant cells	1 (1.0)	-	-	-	-	-	-	-
	Myocardial mineralization	5 (2.8)	1 (3.0)	1 (2.0)	-	-	-	-	-

## Data Availability

The data that support the findings of this study are available on request from the corresponding author.

## Supplementary Materials

The following are available online at https://www.mdpi.com/article/10.3390/pharmaceutics13081269/s1, Figure S1. Phenotypic characterization of the rat and pig ADSCs. Figure S2. Animal weight and food intake during the toxicity study. Tables S1–S4. Irwin’s test for analyzing the general symptomatology in rats. Tables S5–S8. Biochemical and hematological parameters of the sera from the rats. Tables S9–S10. Biochemical parameters of the urine samples from the rats. Tables S11 and S12. Relative weight of the organs in the rats. Table S13. Biochemical parameters assessed in the study.
