# Supplementary material for: Preclinical Evaluation of the Safety and Immunological Action of Allogeneic ADSC-Collagen Scaffolds in the Treatment of Chronic Ischemic Cardiomyopathy
Source: Pharmaceutics. 2021 Aug 17;13(8):1269. doi: 10.3390/pharmaceutics13081269 (PMC8399291; doi:10.3390/pharmaceutics13081269)
Supplement: Supplementary file 1 [file pharmaceutics-13-01269-s001.zip › pharmaceutics-1289323-supplementary.pdf]

pharmaceutics

MDPI

Supplementary Materials: Preclinical Evaluation of the Safety and Immunological Action of Allogeneic ADSC-Collagen Scaffolds in the Treatment of Chronic Ischemic Cardiomyopathy

Ascensión López-Díaz de Cerio, Iñigo Perez-Estenaga, Susana Inoges, Gloria Abizanda, Juan José Gavira, Eduardo Larequi, Enrique Andreu, Saray Rodriguez, Ana Gloria Gil, Verónica Crisostomo, Francisco Miguel Sanchez-Margallo, Javier Bermejo, Blanca Jauregui, Lluis Quintana, Francisco Fernández-Avilés, Beatriz Pelacho and Felipe Prósper

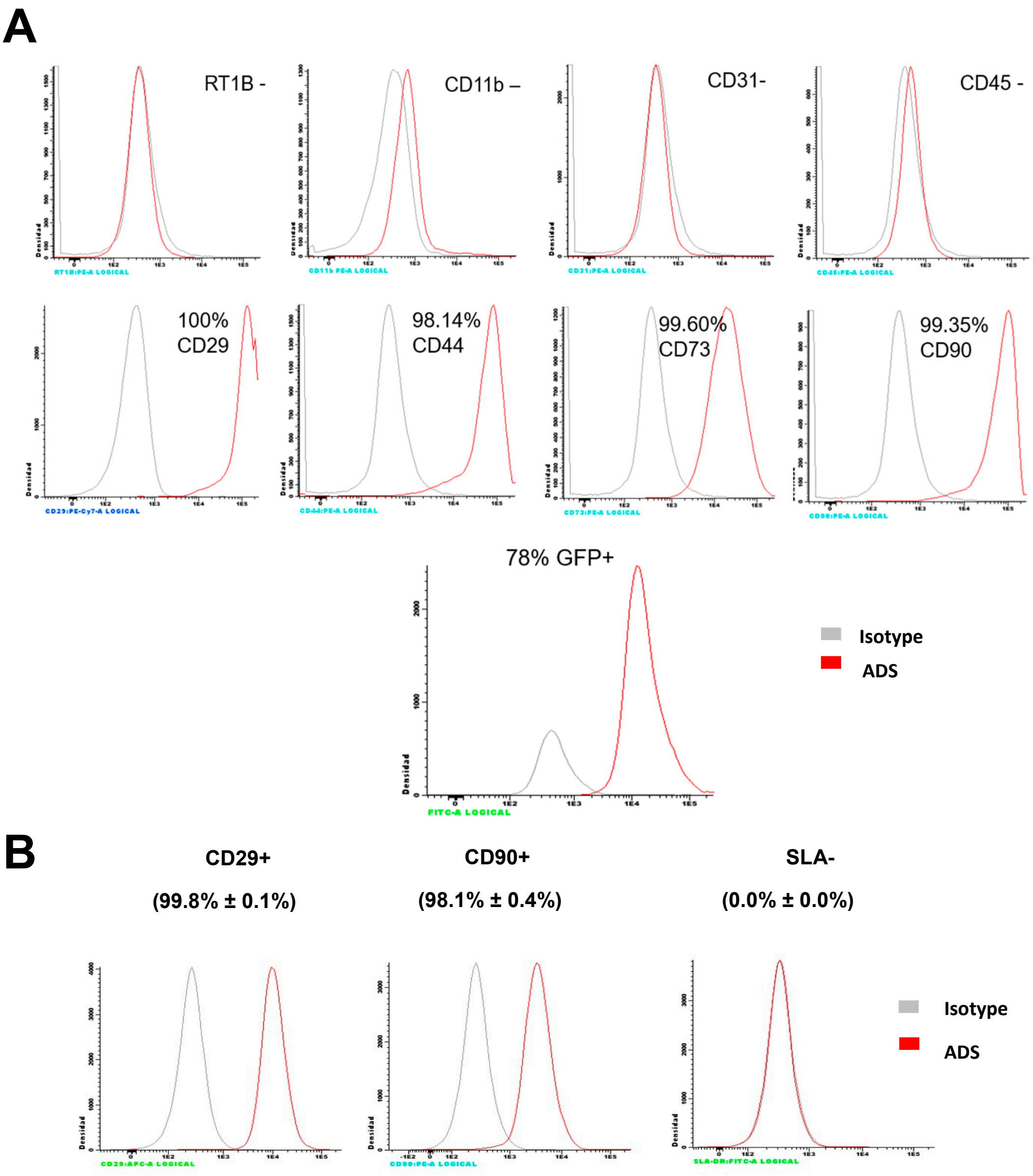

Figure S1. Phenotypic characterization of the rat and pig ADSCs.

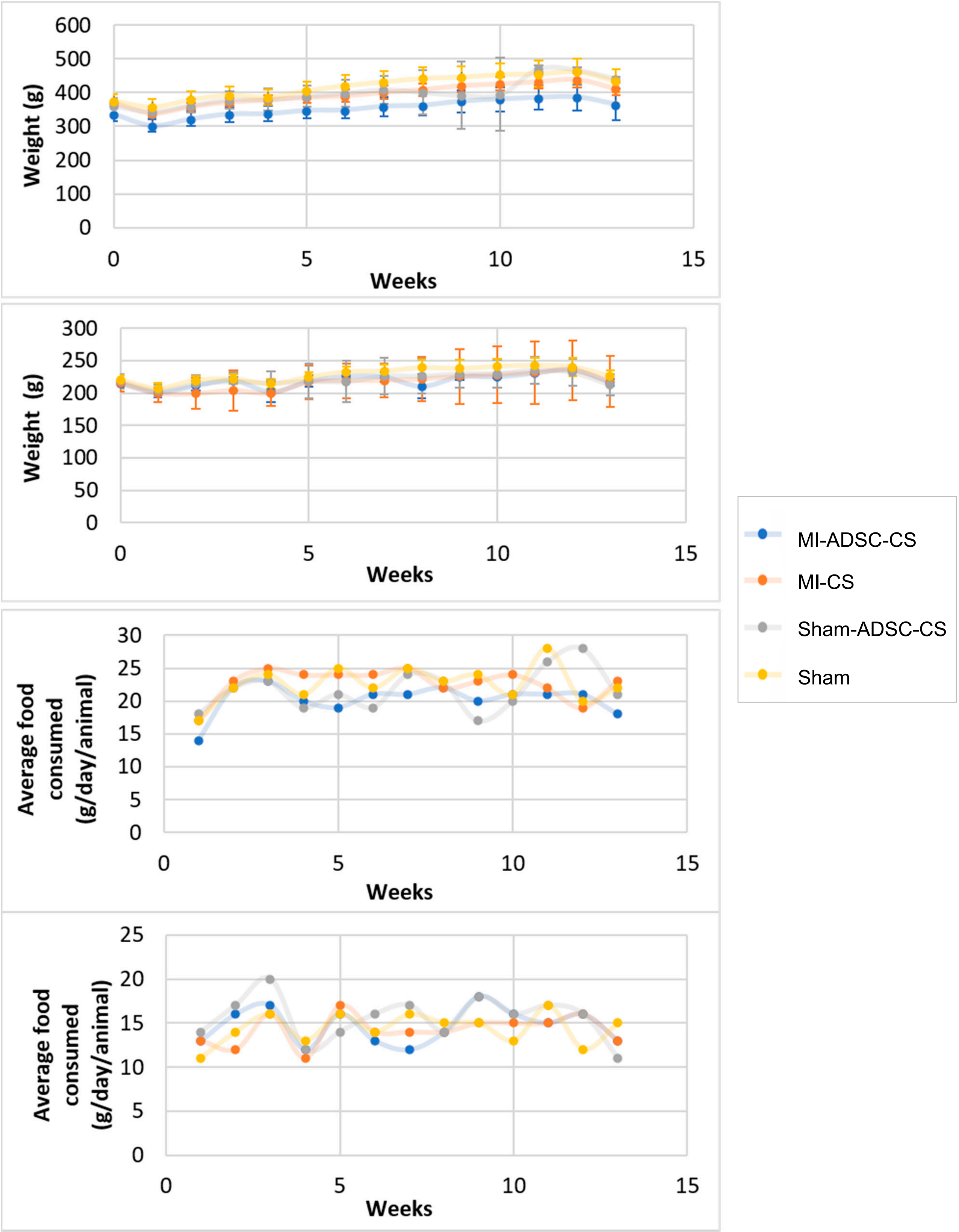

Figure S2. Animal weight and food intake during the toxicity study.

Table S1. Irwin`s test for analysing general symptomatology in the MI-ADSC-CS group. The table shows, for each week, the number of animals in the MI-ADSC-CS group (*n* = 10; 5 males and 5 females) that present the assigned evaluation. This evaluation corresponds to a normal symptomatology for RH-Foxn1^{mu} rats.

Studied parameters / Assigned Evaluation	Observation period (weeks)						Observations
	Week 1	Week 2	Week 3	Week 4	Week 5	Week 6	
**Visual location**	10/10	10/10	10/10	9/10	9/9	9/9	Week 4: Dead Female 10
**Spontaneous activity**	10/10	10/10	10/10	10/10	9/9	9/9	
**Reactivity**	10/10	10/10	10/10	10/10	9/9	9/9	
**Contact response**	10/10	10/10	10/10	10/10	9/9	9/9	
**Pain response**	10/10	10/10	10/10	10/10	9/9	9/9	
**Fright response**	10/10	10/10	10/10	10/10	9/9	9/9	
**Stereotypies**	10/10	10/10	10/10	10/10	9/9	9/9	
**Vocalization**	10/10	10/10	10/10	10/10	9/9	9/9	
**Passivity**	10/10	10/10	10/10	10/10	9/9	9/9	
**Irritability**	10/10	10/10	10/10	10/10	9/9	9/9	
**Fear**	10/10	10/10	10/10	10/10	9/9	9/9	
**Aisle**	10/10	10/10	10/10	10/10	9/9	9/9	
**Corporal position**	10/10	10/10	10/10	10/10	9/9	9/9	
**Press strength**	10/10	10/10	10/10	10/10	9/9	9/9	
**Corporal tone**	10/10	10/10	10/10	10/10	9/9	9/9	
**Members tone**	10/10	10/10	10/10	10/10	9/9	9/9	
**Atrium reflection**	10/10	10/10	10/10	10/10	9/9	9/9	
**Corneal reflex**	10/10	10/10	10/10	10/10	9/9	9/9	
**Ipsilateral reflex**	10/10	10/10	10/10	10/10	9/9	9/9	
**Balance**	10/10	10/10	10/10	9/10	9/9	9/9	Not evaluated in female 10
**Straightening reflex**	10/10	10/10	10/10	9/10	9/9	9/9	Not evaluated in female 10
**Temblors**	10/10	10/10	10/10	10/10	9/9	9/9	
**Contractions**	10/10	10/10	10/10	10/10	9/9	9/9	
**Ataxic march**	10/10	10/10	10/10	10/10	9/9	9/9	
**Hypotonic march**	10/10	10/10	10/10	10/10	9/9	9/9	
**Pelvic elevation**	10/10	10/10	10/10	10/10	9/9	9/9	
**Straub tail**	10/10	10/10	10/10	10/10	9/9	9/9	
**Contortions**	10/10	10/10	10/10	10/10	9/9	9/9	
**Pupillary size**	10/10	10/10	10/10	10/10	9/9	9/9	
**Palpebral opening**	10/10	10/10	9/10	10/10	9/9	9/9	Week 3: Male 4 - eyes semi-closed
**Skin colour**	10/10	10/10	9/10	9/10	8/9	8/9	Week 3-4: Male 2 - mild skin paleness
**Cardiac frequency**	10/10	10/10	10/10	10/10	9/9	9/9	
**Respiratory frequency**	10/10	10/10	10/10	10/10	9/9	9/9	
**Exophthalmos**	10/10	10/10	10/10	10/10	9/9	9/9	
**Urination**	10/10	10/10	10/10	10/10	9/9	9/9	
**Salivation**	10/10	10/10	10/10	10/10	9/9	9/9	
**Piloerection**	10/10	10/10	10/10	10/10	9/9	9/9	
**Diarrheal**	10/10	10/10	10/10	10/10	9/9	9/9	
**Hypothermia**	10/10	10/10	10/10	9/10	9/9	9/9	Week 4: Female 10 - hypothermic

Mortality: Female 10 (Day 29-week 5)

(MI-ADSC-CS group continuation, weeks 7-13)

**Studied parameters / Assigned Evaluation**	**Observation period (weeks)**							**Observations**
	**Week 7**	**Week 8**	**Week 9**	**Week 10**	**Week 11**	**Week 12**	**Week 13**	
**Visual location**	9/9	9/9	8/8	8/8	8/8	8/8	8/8	
**Spontaneous activity**	9/9	9/9	8/8	8/8	8/8	8/8	8/8	
**Reactivity**	9/9	9/9	8/8	8/8	8/8	8/8	8/8	
**Contact response**	9/9	9/9	8/8	8/8	8/8	8/8	8/8	
**Pain response**	9/9	9/9	8/8	8/8	8/8	8/8	8/8	
**Fright response**	9/9	9/9	8/8	8/8	8/8	8/8	8/8	
**Stereotypies**	9/9	9/9	8/8	8/8	8/8	8/8	8/8	
**Vocalization**	9/9	9/9	8/8	8/8	8/8	8/8	8/8	
**Passivity**	9/9	9/9	8/8	8/8	8/8	8/8	8/8	
**Irritability**	9/9	9/9	8/8	8/8	8/8	8/8	8/8	
**Fear**	9/9	9/9	8/8	8/8	8/8	8/8	8/8	
**Aisle**	9/9	9/9	8/8	8/8	8/8	8/8	8/8	
**Corporal position**	9/9	9/9	8/8	8/8	8/8	8/8	8/8	
**Press strength**	9/9	9/9	8/8	8/8	8/8	8/8	8/8	
**Corporal tone**	9/9	9/9	8/8	8/8	8/8	8/8	8/8	
**Members tone**	9/9	9/9	8/8	8/8	8/8	8/8	8/8	
**Atrium reflection**	9/9	9/9	8/8	8/8	8/8	8/8	8/8	
**Corneal reflex**	9/9	9/9	8/8	8/8	8/8	8/8	8/8	
**Ipsilateral reflex**	9/9	9/9	8/8	8/8	8/8	8/8	8/8	
**Balance**	9/9	9/9	8/8	8/8	8/8	8/8	8/8	
**Straightening reflex**	9/9	9/9	8/8	8/8	8/8	8/8	8/8	
**Temblors**	9/9	9/9	8/8	8/8	8/8	8/8	8/8	
**Contractions**	9/9	9/9	8/8	8/8	8/8	8/8	8/8	
**Ataxic march**	9/9	9/9	8/8	8/8	8/8	8/8	8/8	
**Hypotonic march**	9/9	9/9	8/8	8/8	8/8	8/8	8/8	
**Pelvic elevation**	9/9	9/9	8/8	8/8	8/8	8/8	8/8	
**Straub tail**	9/9	9/9	8/8	8/8	8/8	8/8	8/8	
**Contortions**	9/9	9/9	8/8	8/8	8/8	8/8	8/8	
**Pupillary size**	9/9	9/9	8/8	8/8	8/8	8/8	8/8	
**Palpebral opening**	9/9	9/9	8/8	8/8	8/8	8/8	8/8	
**Skin colour**	8/9	8/9	7/8	7/8	8/8	8/8	8/8	Weeks 7-10: Male 2 - mild skin paleness
**Cardiac frequency**	9/9	9/9	8/8	8/8	8/8	8/8	8/8	
**Respiratory frequency**	9/9	9/9	8/8	8/8	8/8	8/8	8/8	
**Exophthalmos**	9/9	9/9	8/8	8/8	8/8	8/8	8/8	
**Urination**	8/9	9/9	8/8	8/8	8/8	8/8	8/8	Week 7: Female 6 - bloody urination
**Salivation**	9/9	9/9	8/8	8/8	8/8	8/8	8/8	
**Piloerection**	9/9	9/9	8/8	8/8	8/8	8/8	8/8	
**Diarrheal**	9/9	9/9	8/8	8/8	8/8	8/8	8/8	
**Hypothermia**	9/9	8/9	8/8	8/8	8/8	8/8	8/8	Week 8: Female 8 - hypothermic
Mortality: Female 10 (Day 29-week 5) and female 8 (Day 57-week 9)								

Table S2. Irwin`s test for analysing general symptomatology in the MI-CS group. The table shows, for each week, the number of animals in the MI-CS group (*n* = 10; 5 males and 5 females) that present the assigned evaluation. This evaluation corresponds to a normal symptomatology for RH-Foxn1rnu rats.

Studied parameters / Assigned Evaluation	Observation period (weeks)						Observations
	Week 1	Week 2	Week 3	Week 4	Week 5	Week 6	
**Visual location**	10/10	10/10	10/10	9/9	8/8	8/8	
**Spontaneous activity**	10/10	10/10	9/10	9/9	8/8	8/8	Week 3: Female 7 - sporadic and slow movements
**Reactivity**	10/10	10/10	10/10	9/9	8/8	8/8	
**Contact response**	10/10	10/10	10/10	9/9	8/8	8/8	
**Pain response**	10/10	10/10	10/10	9/9	8/8	8/8	
**Fright response**	10/10	10/10	10/10	9/9	8/8	8/8	
**Stereotypies**	10/10	10/10	10/10	9/9	8/8	8/8	
**Vocalization**	10/10	10/10	10/10	9/9	8/8	8/8	
**Passivity**	10/10	10/10	10/10	9/9	8/8	8/8	
**Irritability**	10/10	10/10	10/10	9/9	8/8	8/8	
**Fear**	10/10	10/10	10/10	9/9	8/8	8/8	
**Aisle**	10/10	10/10	10/10	9/9	8/8	8/8	
**Corporal position**	10/10	10/10	10/10	9/9	8/8	8/8	
**Press strength**	10/10	10/10	10/10	9/9	8/8	8/8	
**Corporal tone**	10/10	10/10	10/10	9/9	8/8	8/8	
**Members tone**	10/10	10/10	10/10	9/9	8/8	8/8	
**Atrium reflection**	10/10	10/10	10/10	9/9	8/8	8/8	
**Corneal reflex**	10/10	10/10	10/10	9/9	8/8	8/8	
**Ipsilateral reflex**	10/10	10/10	10/10	9/9	8/8	8/8	
**Balance**	10/10	10/10	9/10	9/9	8/8	8/8	No carried out in the Female 7 to avoid excessive effort
**Straightening reflex**	10/10	10/10	9/10	9/9	8/8	8/8	No carried out in the Female 7 to avoid excessive effort
**Temblors**	10/10	10/10	10/10	9/9	8/8	8/8	
**Contractions**	10/10	10/10	10/10	9/9	8/8	8/8	
**Ataxic march**	10/10	10/10	10/10	9/9	8/8	8/8	
**Hypotonic march**	10/10	10/10	10/10	9/9	8/8	8/8	
**Pelvic elevation**	10/10	10/10	10/10	9/9	8/8	8/8	
**Straub tail**	10/10	10/10	10/10	9/9	8/8	8/8	
**Contortions**	10/10	10/10	10/10	9/9	8/8	8/8	
**Pupillary size**	10/10	10/10	10/10	9/9	8/8	8/8	
**Palpebral opening**	10/10	10/10	9/10	9/9	8/8	8/8	Week 3: Female 7 - eyes semi-closed
**Skin colour**	10/10	10/10	10/10	9/9	8/8	8/8	
**Cardiac frequency**	10/10	10/10	9/10	9/9	8/8	8/8	Week 3: Female 7 - tachycardia
**Respiratory frequency**	10/10	10/10	9/10	8/9	7/8	7/8	Week 3: Female 7 - respiratory distress Week 3-6: Female 8 - respiratory distress

**Exophthalmos**	10/10	10/10	10/10	9/9	8/8	8/8	
**Urination**	10/10	10/10	10/10	9/9	8/8	8/8	
**Salivation**	10/10	10/10	10/10	9/9	8/8	8/8	
**Piloerection**	10/10	10/10	10/10	9/9	8/8	8/8	
**Diarrheal**	10/10	10/10	10/10	9/9	8/8	8/8	
**Hypothermia**	10/10	10/10	10/10	9/9	8/8	8/8	
Mortality: Female 7 (Day 24-week 4) and Female 9 (Day 35-week 5)							

(MI-CS group continuation, weeks 7-13)

**Studied parameters / Assigned Evaluation**	**Observation period (weeks)**							**Observations**
	**Week 7**	**Week 8**	**Week 9**	**Week 10**	**Week 11**	**Week 12**	**Week 13**	
**Visual location**	8/8	8/8	8/8	8/8	8/8	8/8	8/8	
**Spontaneous activity**	8/8	8/8	8/8	8/8	8/8	8/8	8/8	
**Reactivity**	8/8	8/8	8/8	8/8	8/8	8/8	8/8	
**Contact response**	8/8	8/8	8/8	8/8	8/8	8/8	8/8	
**Pain response**	8/8	8/8	8/8	8/8	8/8	8/8	8/8	
**Fright response**	8/8	8/8	8/8	8/8	8/8	8/8	8/8	
**Stereotypies**	8/8	8/8	8/8	8/8	8/8	8/8	8/8	
**Vocalization**	8/8	8/8	8/8	8/8	8/8	8/8	8/8	
**Passivity**	8/8	8/8	8/8	8/8	8/8	8/8	8/8	
**Irritability**	8/8	8/8	8/8	8/8	8/8	8/8	8/8	
**Fear**	8/8	8/8	8/8	8/8	8/8	8/8	8/8	
**Aisle**	8/8	8/8	8/8	8/8	8/8	8/8	8/8	
**Corporal position**	8/8	8/8	8/8	8/8	8/8	8/8	8/8	
**Press strength**	8/8	8/8	8/8	8/8	8/8	8/8	8/8	
**Corporal tone**	8/8	8/8	8/8	8/8	8/8	8/8	8/8	
**Members tone**	8/8	8/8	8/8	8/8	8/8	8/8	8/8	
**Atrium reflection**	8/8	8/8	8/8	8/8	8/8	8/8	8/8	
**Corneal reflex**	8/8	8/8	8/8	8/8	8/8	8/8	8/8	
**Ipsilateral reflex**	8/8	8/8	8/8	8/8	8/8	8/8	8/8	
**Balance**	8/8	8/8	8/8	8/8	8/8	8/8	8/8	
**Straightening reflex**	8/8	8/8	8/8	8/8	8/8	8/8	8/8	
**Temblors**	8/8	8/8	8/8	8/8	8/8	8/8	8/8	
**Contractions**	8/8	8/8	8/8	8/8	8/8	8/8	8/8	
**Ataxic march**	8/8	8/8	8/8	8/8	8/8	8/8	8/8	
**Hypotonic march**	8/8	8/8	8/8	8/8	8/8	8/8	8/8	
**Pelvic elevation**	8/8	8/8	8/8	8/8	8/8	8/8	8/8	
**Straub tail**	8/8	8/8	8/8	8/8	8/8	8/8	8/8	
**Contortions**	8/8	8/8	8/8	8/8	8/8	8/8	8/8	
**Pupillary size**	8/8	8/8	8/8	8/8	8/8	8/8	8/8	
**Palpebral opening**	8/8	8/8	8/8	8/8	8/8	8/8	8/8	
**Skin colour**	8/8	8/8	8/8	8/8	8/8	8/8	8/8	
**Cardiac frequency**	8/8	8/8	8/8	8/8	8/8	8/8	8/8	
**Respiratory frequency**	7/8	7/8	7/8	7/8	7/8	7/8	8/8	Week 7-12: Female 8 - respiratory distress
**Exophthalmos**	8/8	8/8	8/8	8/8	8/8	8/8	8/8	
**Urination**	8/8	8/8	8/8	8/8	8/8	8/8	8/8	
**Salivation**	8/8	8/8	8/8	8/8	8/8	8/8	8/8	

**Piloerection**	8/8	8/8	8/8	8/8	8/8	8/8	8/8	
**Diarrheal**	8/8	8/8	8/8	8/8	8/8	8/8	8/8	
**Hypothermia**	8/8	8/8	8/8	8/8	8/8	8/8	8/8	
Mortality: Female 7 (Day 24-week 4) and Female 9 (Day 35-week 5)								

Table S3. Irwin`s test for analysing general symptomatology in the Sham-ADSC-CS group. The table shows, for each week, the number of animals in the Sham-ADSC-CS group (n = 10; 5 males and 5 females) that present the assigned evaluation. This evaluation corresponds to a normal symptomatology for RH-Foxn1rnu rats.

Studied parameters / Assigned Evaluation	Observation period (weeks)						Observations
	Week 1	Week 2	Week 3	Week 4	Week 5	Week 6	
**Visual location**	10/10	10/10	10/10	10/10	9/9	8/8	
**Spontaneous activity**	10/10	10/10	10/10	10/10	9/9	8/8	
**Reactivity**	10/10	10/10	10/10	10/10	9/9	8/8	
**Contact response**	10/10	10/10	10/10	10/10	9/9	8/8	
**Pain response**	10/10	10/10	10/10	10/10	9/9	8/8	
**Fright response**	10/10	10/10	10/10	10/10	9/9	8/8	
**Stereotypies**	10/10	10/10	10/10	10/10	9/9	8/8	
**Vocalization**	10/10	10/10	10/10	10/10	9/9	8/8	
**Passivity**	10/10	10/10	10/10	10/10	9/9	8/8	
**Irritability**	10/10	10/10	10/10	10/10	9/9	8/8	
**Fear**	10/10	10/10	10/10	10/10	9/9	8/8	
**Aisle**	10/10	10/10	10/10	10/10	9/9	8/8	
**Corporal position**	10/10	10/10	10/10	10/10	9/9	8/8	
**Press strength**	10/10	10/10	10/10	10/10	9/9	8/8	
**Corporal tone**	10/10	10/10	10/10	10/10	9/9	8/8	
**Members tone**	10/10	10/10	10/10	10/10	9/9	8/8	
**Atrium reflection**	10/10	10/10	10/10	10/10	9/9	8/8	
**Corneal reflex**	10/10	10/10	10/10	10/10	9/9	8/8	
**Ipsilateral reflex**	10/10	10/10	10/10	10/10	9/9	8/8	
**Balance**	10/10	10/10	10/10	10/10	9/9	8/8	
**Straightening reflex**	10/10	10/10	10/10	10/10	9/9	8/8	
**Temblors**	10/10	10/10	10/10	10/10	9/9	8/8	
**Contractions**	10/10	10/10	10/10	10/10	9/9	8/8	
**Ataxic march**	10/10	10/10	10/10	10/10	9/9	8/8	
**Hypotonic march**	10/10	10/10	10/10	10/10	9/9	8/8	
**Pelvic elevation**	10/10	10/10	10/10	10/10	9/9	8/8	
**Straub tail**	10/10	10/10	10/10	10/10	9/9	8/8	
**Contortions**	10/10	10/10	10/10	10/10	9/9	8/8	
**Pupillary size**	10/10	10/10	10/10	10/10	9/9	8/8	
**Palpebral opening**	10/10	10/10	10/10	10/10	9/9	8/8	
**Skin colour**	10/10	10/10	10/10	10/10	9/9	8/8	
**Cardiac frequency**	10/10	10/10	10/10	10/10	9/9	8/8	
**Respiratory frequency**	10/10	10/10	10/10	10/10	9/9	8/8	
**Exophthalmos**	10/10	10/10	10/10	10/10	9/9	8/8	
**Urination**	10/10	10/10	10/10	10/10	9/9	8/8	
**Salivation**	10/10	10/10	10/10	10/10	9/9	8/8	

**Piloerection**	10/10	10/10	10/10	10/10	9/9	8/8	
**Diarrheal**	10/10	10/10	10/10	10/10	8/9	8/8	Week 4: Male 4 - diarrhea
**Hypothermia**	10/10	10/10	10/10	10/10	8/9	7/8	Week 5: Male 4 - hypothermia<br>Week 6: Female 8 - hypothermia
Mortality: Male 5 (Day 28-week 5) and Male 4 (Day 41-week 6)							

(Sham-ADSC-CS group continuation, weeks 7-13)

**Studied parameters / Assigned Evaluation**	**Observation period (weeks)**							**Observations**
	**Week 7**	**Week 8**	**Week 9**	**Week 10**	**Week 11**	**Week 12**	**Week 13**	
**Visual location**	8/8	8/8	8/8	7/7	7/7	7/7	7/7	
**Spontaneous activity**	8/8	8/8	8/8	7/7	7/7	7/7	7/7	
**Reactivity**	8/8	8/8	8/8	7/7	7/7	7/7	7/7	
**Contact response**	8/8	8/8	8/8	7/7	7/7	7/7	7/7	
**Pain response**	8/8	8/8	8/8	7/7	7/7	7/7	7/7	
**Fright response**	8/8	8/8	8/8	7/7	7/7	7/7	7/7	
**Stereotypies**	8/8	8/8	8/8	7/7	7/7	7/7	7/7	
**Vocalization**	8/8	8/8	8/8	7/7	7/7	7/7	7/7	
**Passivity**	8/8	8/8	8/8	7/7	7/7	7/7	7/7	
**Irritability**	8/8	8/8	8/8	7/7	7/7	7/7	7/7	
**Fear**	8/8	8/8	8/8	7/7	7/7	7/7	7/7	
**Aisle**	8/8	8/8	8/8	7/7	7/7	7/7	7/7	
**Corporal position**	8/8	8/8	8/8	7/7	7/7	7/7	7/7	
**Press strength**	8/8	8/8	8/8	7/7	7/7	7/7	7/7	
**Corporal tone**	8/8	8/8	8/8	7/7	7/7	7/7	7/7	
**Members tone**	8/8	8/8	8/8	7/7	7/7	7/7	7/7	
**Atrium reflection**	8/8	8/8	8/8	7/7	7/7	7/7	7/7	
**Corneal reflex**	8/8	8/8	8/8	7/7	7/7	7/7	7/7	
**Ipsilateral reflex**	8/8	8/8	8/8	7/7	7/7	7/7	7/7	
**Balance**	8/8	8/8	8/8	7/7	7/7	7/7	7/7	
**Straightening reflex**	8/8	8/8	8/8	7/7	7/7	7/7	7/7	
**Temblors**	8/8	8/8	8/8	7/7	7/7	7/7	7/7	
**Contractions**	8/8	8/8	8/8	7/7	7/7	7/7	7/7	
**Ataxic march**	8/8	8/8	8/8	7/7	7/7	7/7	7/7	
**Hypotonic march**	8/8	8/8	8/8	7/7	7/7	7/7	7/7	
**Pelvic elevation**	8/8	8/8	8/8	7/7	7/7	7/7	7/7	
**Straub tail**	8/8	8/8	8/8	7/7	7/7	7/7	7/7	
**Contortions**	8/8	8/8	8/8	7/7	7/7	7/7	7/7	
**Pupillary size**	8/8	8/8	8/8	7/7	7/7	7/7	7/7	
**Palpebral opening**	8/8	8/8	8/8	7/7	7/7	7/7	7/7	
**Skin colour**	8/8	8/8	8/8	7/7	7/7	7/7	7/7	
**Cardiac frequency**	8/8	8/8	8/8	7/7	7/7	7/7	7/7	
**Respiratory frequency**	8/8	8/8	8/8	7/7	7/7	7/7	7/7	
**Exophthalmos**	8/8	8/8	8/8	7/7	7/7	7/7	7/7	
**Urination**	8/8	8/8	8/8	7/7	7/7	7/7	7/7	
**Salivation**	8/8	8/8	8/8	7/7	7/7	7/7	7/7	
**Piloerection**	8/8	8/8	8/8	7/7	7/7	7/7	7/7	
**Diarrheal**	8/8	8/8	8/8	7/7	7/7	7/7	7/7	

**Hypothermia**	8/8	8/8	8/8	7/7	7/7	7/7	7/7	
Mortality: Male 5 (Day 28-week 5); Male 4 (Day 41-week 6) and Male Reserve 1 (Day 65-week 10)								

Table S4. Irwin`s test for analysing general symptomatology in the Sham Group. The table shows, for each week, the number of animals in the Sham group (n = 10; 5 males and 5 females) that present the assigned evaluation. This evaluation corresponds to a normal symptomatology for RH-Foxn1rnu rats.

**Studied parameters / Assigned Evaluation**	**Observation period (weeks)**						**Observations**
	**Week 1**	**Week 2**	**Week 3**	**Week 4**	**Week 5**	**Week 6**	
**Visual location**	10/10	10/10	10/10	10/10	10/10	10/10	
**Spontaneous activity**	10/10	10/10	10/10	10/10	10/10	10/10	
**Reactivity**	10/10	10/10	10/10	10/10	10/10	10/10	
**Contact response**	10/10	10/10	10/10	10/10	10/10	10/10	
**Pain response**	10/10	10/10	10/10	10/10	10/10	10/10	
**Fright response**	10/10	10/10	10/10	10/10	10/10	10/10	
**Stereotypies**	10/10	10/10	10/10	10/10	10/10	10/10	
**Vocalization**	10/10	10/10	10/10	10/10	10/10	10/10	
**Passivity**	10/10	10/10	10/10	10/10	10/10	10/10	
**Irritability**	10/10	10/10	10/10	10/10	10/10	10/10	
**Fear**	10/10	10/10	10/10	10/10	10/10	10/10	
**Aisle**	10/10	10/10	10/10	10/10	10/10	10/10	
**Corporal position**	10/10	10/10	10/10	10/10	10/10	10/10	
**Press strength**	10/10	10/10	10/10	10/10	10/10	10/10	
**Corporal tone**	10/10	10/10	10/10	10/10	10/10	10/10	
**Members tone**	10/10	10/10	10/10	10/10	10/10	10/10	
**Atrium reflection**	10/10	10/10	10/10	10/10	10/10	10/10	
**Corneal reflex**	10/10	10/10	10/10	10/10	10/10	10/10	
**Ipsilateral reflex**	10/10	10/10	10/10	10/10	10/10	10/10	
**Balance**	10/10	10/10	10/10	10/10	10/10	10/10	
**Straightening reflex**	10/10	10/10	10/10	10/10	10/10	10/10	
**Temblors**	10/10	10/10	10/10	10/10	10/10	10/10	
**Contractions**	10/10	10/10	10/10	10/10	10/10	10/10	
**Ataxic march**	10/10	10/10	10/10	10/10	10/10	10/10	
**Hypotonic march**	10/10	10/10	10/10	10/10	10/10	10/10	
**Pelvic elevation**	10/10	10/10	10/10	10/10	10/10	10/10	
**Straub tail**	10/10	10/10	10/10	10/10	10/10	10/10	
**Contortions**	10/10	10/10	10/10	10/10	10/10	10/10	
**Pupillary size**	10/10	10/10	10/10	10/10	10/10	10/10	
**Palpebral opening**	10/10	10/10	10/10	10/10	10/10	10/10	
**Skin colour**	10/10	10/10	10/10	10/10	10/10	10/10	
**Cardiac frequency**	10/10	10/10	10/10	10/10	10/10	10/10	
**Respiratory frequency**	10/10	10/10	10/10	10/10	10/10	10/10	
**Exophthalmos**	10/10	10/10	10/10	10/10	10/10	10/10	
**Urination**	10/10	10/10	10/10	10/10	10/10	10/10	
**Salivation**	10/10	10/10	10/10	10/10	10/10	10/10	
**Piloerection**	10/10	10/10	10/10	10/10	10/10	10/10	

**Diarrheal**	10/10	10/10	10/10	10/10	10/10	10/10	
**Hypothermia**	10/10	10/10	10/10	10/10	10/10	10/10	

(Sham Group continuation, weeks 7-13)

**Studied parameters / Assigned Evaluation**	**Observation period (weeks)**							**Observations**
	**Week 7**	**Week 8**	**Week 9**	**Week 10**	**Week 11**	**Week 12**	**Week 13**	
**Visual location**	10/10	10/10	10/10	10/10	10/10	10/10	10/10	
**Spontaneous activity**	10/10	10/10	10/10	10/10	10/10	10/10	10/10	
**Reactivity**	10/10	10/10	10/10	10/10	10/10	10/10	10/10	
**Contact response**	10/10	10/10	10/10	10/10	10/10	10/10	10/10	
**Pain response**	10/10	10/10	10/10	10/10	10/10	10/10	10/10	
**Fright response**	10/10	10/10	10/10	10/10	10/10	10/10	10/10	
**Stereotypies**	10/10	10/10	10/10	10/10	10/10	10/10	10/10	
**Vocalization**	10/10	10/10	10/10	10/10	10/10	10/10	10/10	
**Passivity**	10/10	10/10	10/10	10/10	10/10	10/10	10/10	
**Irritability**	10/10	10/10	10/10	10/10	10/10	10/10	10/10	
**Fear**	10/10	10/10	10/10	10/10	10/10	10/10	10/10	
**Aisle**	10/10	10/10	10/10	10/10	10/10	10/10	10/10	
**Corporal position**	10/10	10/10	10/10	10/10	10/10	10/10	10/10	
**Press strength**	10/10	10/10	10/10	10/10	10/10	10/10	10/10	
**Corporal tone**	10/10	10/10	10/10	10/10	10/10	10/10	10/10	
**Members tone**	10/10	10/10	10/10	10/10	10/10	10/10	10/10	
**Atrium reflection**	10/10	10/10	10/10	10/10	10/10	10/10	10/10	
**Corneal reflex**	10/10	10/10	10/10	10/10	10/10	10/10	10/10	
**Ipsilateral reflex**	10/10	10/10	10/10	10/10	10/10	10/10	10/10	
**Balance**	10/10	10/10	10/10	10/10	10/10	10/10	10/10	
**Straightening reflex**	10/10	10/10	10/10	10/10	10/10	10/10	10/10	
**Temblors**	10/10	10/10	10/10	10/10	10/10	10/10	10/10	
**Contractions**	10/10	10/10	10/10	10/10	10/10	10/10	10/10	
**Ataxic march**	10/10	10/10	10/10	10/10	10/10	10/10	10/10	
**Hypotonic march**	10/10	10/10	10/10	10/10	10/10	10/10	10/10	
**Pelvic elevation**	10/10	10/10	10/10	10/10	10/10	10/10	10/10	
**Straub tail**	10/10	10/10	10/10	10/10	10/10	10/10	10/10	
**Contortions**	10/10	10/10	10/10	10/10	10/10	10/10	10/10	
**Pupillary size**	10/10	10/10	10/10	10/10	10/10	10/10	10/10	
**Palpebral opening**	10/10	10/10	10/10	10/10	10/10	10/10	10/10	
**Skin colour**	10/10	10/10	10/10	10/10	10/10	10/10	10/10	
**Cardiac frequency**	10/10	10/10	10/10	10/10	10/10	10/10	10/10	
**Respiratory frequency**	10/10	10/10	10/10	10/10	10/10	10/10	10/10	
**Exophthalmos**	10/10	10/10	10/10	10/10	10/10	10/10	10/10	
**Urination**	10/10	10/10	10/10	10/10	10/10	10/10	10/10	
**Salivation**	10/10	10/10	10/10	10/10	10/10	10/10	10/10	
**Piloerection**	10/10	10/10	10/10	10/10	10/10	10/10	10/10	
**Diarrheal**	10/10	10/10	10/10	10/10	10/10	10/10	10/10	
**Hypothermia**	10/10	10/10	10/10	10/10	10/10	10/10	10/10	

Table S5. Serum biochemistry in male rats. Ordered by experimental group, the descriptive statistics of centralization and dispersion (mean and standard deviation) and the statistical significance obtained by the one-way non-parametric ANOVA (Kruskal-Wallis test, according to the levels of significance: (*) p <0.05 and (**) p <0.01 are included). MI-ADSC-CS, MI-CS and Sham-ADSC-CS vs. Sham control group. Analysis was performed in 3 animals per each experimental group and sex for day 2 and 10 post-implant, and in 5 animals for day 28 and 90 post-implant.

MALE	Time of analysis	Albumin (g/dl)	Urea (mg/dl)	AST (U/l)	ALT (U/l)	ALP (U/l)	T. Bilirubin (mg/dl)	Cholesterol (mg/dl)	Glucose (mg/ml)	Creatinine (mg/dl)	T. Proteins (g/dl)	CPK (U/l)	GGT (U/l)	Chloro (mmol/l)	Potassium (mmol/l)	Sodium (mmol/l)	Globuline (g/dl)
MI-ADSC-CS	**Day 2**	3.1 ± 0.1	36 ± 5	195 ± 8	35 ± 2	81 ± 12	0.09 ± 0.01	74 ± 4	97 ± 9	0.30 ± 0.02	5.2 ± 0.2	1058 ± 474	0 ± 0	96.4 ± 0.9	4.33 ± .05	132 ± 5	2.1 ± 0.2
	**Day 10**	3.2 ± 0.2	34 ± 6	101 ± 17	16 ± 2	58 ± 4	0.09 ± 0.01	69 ± 5	66 ± 13	0.34 ± 0.06	4.9 ± 0.4	604 ± 155	0 ± 0	98.6 ± 5.8	4.44 ± 0.23	140 ± 11	1.7 ± 0.2
	**Day 28**	3.7 ± 0.1	31 ± 5	72 ± 2	31 ± 5	78 ± 2	0.10 ± 0.02	92 ± 1	189 ± 9	0.35 ± 0.04	5.7 ± 0.1	345 ± 95	0 ± 0	92.9 ± 1.3	4.75 ± 0.21	142 ± 1(*)	2.0 ± 0.1
	**Day 90**	3.6 ± 0.2	37 ± 5	96 ± 8	21 ± 3	51 ± 11	0.08 ± 0.01	89 ± 8	107 ± 13	0.39 ± 0.02	5.3 ± 0.4	529 ± 201	0 ± 0	90.9 ± 1.2	4.32 ± 0.27	126 ± 2	1.7 ± 0.3
MI-CS	**Day 2**	3.3 ± 0.1	37 ± 2	108 ± 13	19 ± 3	78 ± 10	0.09 ± 0.01	78 ± 6	107 ± 13	0.36 ± 0.03	5.6 ± 0.1	537 ± 128	0 ± 0	93.3 ± 1.1	4.19 ± 0.12	115 ± 11(*)	2.2 ± 0.1
	**Day 10**	3.6 ± 0.2	37 ± 3	82 ± 8	17 ± 3	66 ± 4	0.10 ± 0.01	78 ± 10	113 ± 14	0.35 ± 0.03	5.2 ± 0.1	412 ± 102	0 ± 0	98.8 ± 5.8	4.28 ± 0.18	136 ± 9	1.6 ± 0.1
	**Day 28**	3.5 ± 0.1	25 ± 2	70 ± 9	32 ± 7	70 ± 12	0.08 ± 0.01	87 ± 9	223 ± 29	0.34 ± 0.03	5.4 ± 0.1	309 ± 53	0 ± 0	94.7 ± 0.2(**)	4.45 ± 0.39	146 ± 6(*)	1.9 ± 0
	**Day 90**	3.8 ± 0.1	36 ± 2	82 ± 23	21 ± 2	51 ± 9	0.10 ± 0.02(**)	92 ± 8	120 ± 11	0.40 ± 0.03	5.6 ± 0.2	541 ± 454	0 ± 0	89.2 ± 4.9	3.93 ± 0.40	120 ± 9	1.8 ± 0
Sham-ADSC-CS	**Day 2**	3.1 ± 0.2	44 ± 6	183 ± 36	33 ± 7	85 ± 41	0.12 ± 0.04	92 ± 16	127 ± 12	0.37 ± 0.03	5.4 ± 0.2	1291 ± 685	1 ± 1	89.0 ± 0.6(*)	3.99 ± 0.22	128 ± 4	2.3 ± 0.1
	**Day 10**	3.6 ± 0.4	32 ± 4	107 ± 8	21 ± 2	59 ± 5	0.10 ± 0.03	82 ± 4	101 ± 26	0.35 ± 0.04	5.3 ± 0.3	830 ± 462	0 ± 0	99.8 ± 0.8	4.48 ± 0.42	139 ± 1	1.7 ± 0.2
	**Day 28**	3.5 ± 0.2	30 ± 6	81 ± 27	39 ± 21	65 ± 13	0.11 ± 0.02	93 ± 7	208 ± 28	0.30 ± 0.04	5.2 ± 0.2	337 ± 184	0 ± 0	91.0 ± 0.8	4.45 ± 0.25	131 ± 2	1.7 ± 0(*)
	**Day 90**	3.7 ± 0.1	35 ± 6	80 ± 29	17 ± 4	41 ± 3	0.04 ± 0.01	83 ± 0	132 ± 16	0.37 ± 0.04	5.5 ± 0.1	757 ± 710	0 ± 0	92.1 ± 2.6	4.04 ± 0.11	135 ± 4	1.8 ± 0
Sham	**Day 2**	3.1 ± 0.3	38 ± 3	129 ± 40	38 ± 21	59 ± 5	0.12 ± 0.02	85 ± 19	116 ± 5	0.30 ± 0.02	5.1 ± 0.5	916 ± 885	0 ± 0	100.0 ± 0.5	4.56 ± 0.44	139 ± 1	2.1 ± 0.2
	**Day 10**	3.7 ± 0.1	34 ± 6	107 ± 12	22 ± 2	56 ± 6	0.11 ± 0.01	77 ± 7	106 ± 3	0.38 ± 0.02	5.3 ± 0.2	379 ± 127	0 ± 0	98.2 ± 0.3	4.72 ± 0.21	124 ± 26	1.6 ± 0.2
	**Day 28**	3.6 ± 0.1	32 ± 3	122 ± 20	48 ± 13	64 ± 13	0.07 ± 0.01	78 ± 5	194 ± 32	0.29 ± 0.02	5.5 ± 0.1	520 ± 375	0 ± 0	87.3 ± 0.8	4.64 ± 0.21	127 ± 2	1.9 ± 0
	**Day 90**	3.9 ± 0.4	37 ± 9	96 ± 17	20 ± 7	43 ± 6	0.03 ± 0.02	90 ± 8	127 ± 34	0.41 ± 0.06	5.9 ± 0.7	694 ± 675	1 ± 2	93.0 ± 6.6	4.39 ± 0.38	143 ± 18	2.1 ± 0.5

Table S6. Serum biochemistry in female rats. Ordered by experimental group, the descriptive statistics of centralization and dispersion (mean and standard deviation) and the statistical significance obtained by the one-way non-parametric ANOVA (Kruskal-Wallis test, according to the levels of significance: (*) *p* <0.05 and (**) *p* <0.01 are included). MI-ADSC-CS, MI-CS and Sham-ADSC-CS vs. Sham control group. Analysis was performed in 3 animals per each experimental group and sex for day 2 and 10 post-implant and in 5 animals for day 28 and 90 post-implant.

FEMALE	Time of analysis	Albumin (g/dl)	Urea (mg/dl)	AST (U/l)	ALT (U/l)	ALP (U/l)	T. Bilirubin (mg/dl)	Cholesterol (mg/dl)	Glucose (mg/ml)	Creatinine (mg/dl)	T. Proteins (g/dl)	CPK (U/l)	GGT (U/l)	Chloro (mmol/l)	Potassium (mmol/l)	Sodium (mmol/l)	Globulin (g/dl)
MI-ADSC-CS	Day 2	2.7 ± 0.2	37 ± 2	180 ± 22	33 ± 7	54 ± 3	0.11 ± 0.03	65 ± 7	75 ± 5(*)	0.30 ± 0.05	4.5 ± 0.4	690 ± 179	0 ± 0	98.5 ± 1.0	4.39 ± 0.16	136 ± 10	1.8 ± 0.2
	Day 10	3.3 ± 0.3	48 ± 12	118 ± 9	16 ± 3	37 ± 6	0.09 ± 0	74 ± 13	70 ± 7	0.35 ± 0.03	4.6 ± 0.3	670 ± 156	0 ± 0	98.0 ± 4.0	4.71 ± 0.42	134 ± 4	1.8 ± 0.2
	Day 28	4.1 ± 0.1	47 ± 4	79 ± 7	20 ± 5	46 ± 1	0.06 ± 0	93 ± 4	102 ± 26	0.41 ± 0.04	5.6 ± 0.1	433 ± 37	0 ± 0	100.4 ± 0.4(*)	4.09 ± 0.41	147 ± 2	1.5 ± 0.0
	Day 90	3.9 ± 0.3	46 ± 4	97 ± 16	17 ± 3(*)	37 ± 8(*)	0.12 ± 0.03	92 ± 19	92 ± 4	0.41 ± 0.06	5 ± 0.3	522 ± 144	0 ± 0	99.3 ± 2.2	4.80 ± 0.17	133 ± 1	1.6 ± 0.4
MI-CS	Day 2	3.2 ± 0.5	43 ± 8	117 ± 20	18 ± 1(*)	45 ± 4	0.08 + 0.01	87 ± 20	88 + 2	0.33 ± 0.06	5.1 ± 0.6	406 ± 157	0 ± 0	93.9 ± 0.2	4.26 ± 0.27	130 ± 11	1.9 ± 0.2
	Day 10	3.7 ± 0,3	47 ± 9	65 ± 6	12 ± 1	39 ± 1	0.06 ± 0.01	98 ± 15	95 ± 6	0.41 ± 0,05	5.2 ± 0.4	216 ± 87	0 ± 0	96.3 ± 1.4	4.01 ± 0.07	132 ± 3(*)	1.4 ± 0.1
	Day 28	3.9 ± 0.4	46 ± 10	131 ± 102	46 ± 56	77 ± 36	0.11 ± 0.06	107 ± 21	166 ± 164	0.43 ± 0.04(*)	6 ± 0.3	268 ± 37	3 ± 6(*)	97 ± 4.7(*)	4.34 ± 0.06	150 ± 6(*)	1.8 ± 0.1(*)
	Day 90	3.9 ± 0.3	38 ± 3	90 ± 20	21 ± 6(*)	33 ± 15(*)	0.15 ± 0.04(*)	98 ± 14	119 ± 33	0.43 ± 0.01	5.4 ± 0.5	391 ± 37	1 ± 2	100 ± 0.8	3.62 ± 0.55	134 ± 1	1.5 ± 0.2
Sham-ADSC-CS	Day 2	3.2 ± 0.5	43 ± 6	166 ± 9	27 ± 2	53 ± 14	0.10 ± 0.03	83 ± 6	104 ± 20	0.26 ± 0.03	4.9 ± 0.6	718 ± 231	1 ± 2	92.1 ± 0.4(*)	3.68 ± 0.13	130 ± 2	1.7 ± 0.2
	Day 10	3.5 ± 0.2	49 ± 2	95 ± 10	14 ± 2	38 ± 3	0.09 ± 0.01	78 ± 3	84 ± 16	0.39 ± 0.06	5.0 ± 0.2	525 ± 180	0 ± 0	101.9 ± 0.7	4.38 ± 0.20	137 ± 3	1.5 ± 0.0
	Day 28	3.8 ± 0.2	32 ± 5	92 ± 14	29 ± 3	42 ± 3	0.08 ± 0.03	88 ± 10	197 ± 10	0.37 ± 0.01	5.3 ± 0.3	546 ± 115	0 ± 0	90 ± 0.7(*)	3.85 ± 0.30	129 ± 3	1.5 ± 0.1
	Day 90	3.9 ± 0.4	44 ± 12	384 ± 642(*)	199 ± 404	27 ± 12	0.08 ± 0.04	65 ± 12	68 ± 20	0.41 ± 0.06	5 ± 0.5	523 ± 136	1 ± 1	94.1 ± 7.9	3.76 ± 0.27	138 ± 11	1.3 ± 0.2
Sham	Day 2	2.9 ± 0.3	44 ± 7	111 ± 17	25 ± 3	29 ± 2	0.09 ± 0.03	90 ± 13	112 ± 11	0.29 ± 0.01	4.6 ± 0.3	363 ± 182	0 ± 0	102.4 ± 1.3	4.38 ± 0.42	137 ± 2	1.7 ± 0.1
	Day 10	3.6 ± 0.2	46 ± 2	100 ± 17	14 ± 4	34 ± 2	0.10 ± 0.03	87 ± 8	95 ± 8	0.40 ± 0.02	5.2 ± 0.2	689 ± 247	0 ± 0	99.1 ± 0.6	4.26 ± 0.46	146 ± 3	1.6 ± 0.2
	Day 28	3.9 ± 0.4	29 ± 2	85 ± 22	37 ± 6	46 ± 6	0.07 ± 0.02	83 ± 8	160 ± 9	0.32 ± 0.05	5.4 ± 0.4	372 ± 343	0 ± 0	88.1 ± 0.9	3.98 ± 0.25	126 ± 2	1.4 ± 0.2
	Day 90	4.5 ± 0.4	43 ± 6	67 ± 10	13 ± 1	19 ± 2	0.05 ± 0.03	98 ± 6	111 ± 11	0.43 ± 0.02	6.0 ± 0.4	337 ± 163	1 ± 0	96.2 ± 1.7	3.87 ± 0.18	142 ± 11	1.5 ± 0.1

Table S7. Haematological parameters in male rats. Ordered by experimental group, the descriptive statistics of centralization and dispersion (mean and standard deviation) and the statistical significance obtained by one-way non-parametric ANOVA (Kruskal-Wallis test, according to the levels of significance: (*) p <0.05 and (**) p <0.01 are included). MI-ADSC-CS, MI-CS and Sham-ADSC-CS vs. Sham control group. Analysis was performed in 3 animals per each experimental group and sex for day 2 and 10 post-implant and in 5 animals for day 28 and 90 post-implant. Reticulocytes were not determined (n.d.) on Day 28.

MALE	Time of analysis	RBC ($\times 10^6$cell/ml)	WBC ($\times 10^3$cell/ml)	Haemoglobin (g/dl)	Hematocrit (%)	MCV (fL)	MCH (pg)	MCHC (g/dl)	Platelet ($\times 10^3$cell /ml)	Reticulocytes (%)
MI-ADSC-CS	Day 2	7.59 ± 0.21	7.42 ± 1.40	13.4 ± 0.2	39.9 ± 0.3	52.6 ± 1.1	17.6 ± 0.2	33.5 ± 0.3	879 ± 86	3 ± 1
	Day 10	8.39 ± 0.27	8.90 ± 2.13	14.6 ± 0.5	42.6 ± 0.8	50.8 ± 0.8	17.4 ± 0.3	34.3 ± 0.6	1034 ± 40	2 ± 0
	Day 28	9.13 ± 0.27	7.08 ± 0.41	16.0 ± 0.3(*)	45.9 ± 1.1	50.2± 0.5(*)	17.6 ± 0.2	34.9 ± 0.4	1160 ± 96	n.d.
	Day 90	8.00 ± 1.55	6.23 ± 1.26	12.8 ± 4.1	36.9 ± 10.6	45.3 ± 5.6	15.5 ± 2.6	34.2 ± 1.7	1129 ± 28	6 ± 8
MI-CS	Day 2	7.45 ± 1.50	6.50 ± 1.4	14.4 ± 0	38.9 ± 6.3	52.5 ± 2.3	19.8 ± 3.5	37.5 ± 4.7	922 ± 209	2 ± 1
	Day 10	8.14 ± 0.02(*)	9.06 ± 2.20	14.5 ± 0.1	41.3 ± 0.9	50.8 ± 1.2	17.9 ± 0.1	35.2 ± 0.7	942 ± 116	2 ± 0
	Day 28	8.87 ± 0.16	7.12 ± 0.27	15.2 ± 0.2	43.9 ± 0.7	49.5 ± 0.1(**)	17.2 ± 0.1	34.7 ± 0.2	1071 ± 68	n.d.
	Day 90	8.81 ± 0.24	6.31 ± 0.70	14.7 ± 0.6	42.8 ± 0.7	48.6 ± 0.5	16.7 ± 0.5	34.4 ± 0.9	1112 ± 51	1 ± 1
Sham-ADSC-CS	Day 2	8.21 ± 0.19	9.79 ± 2.86	14.4 ± 0.4	42.1 ± 0.7	51.2 ± 0.4	17.5 ± 0.1	34.3 ± 0.4	786 ± 367	1 ± 0
	Day 10	8.28 ± 0.02	12.22 ± 3.37	14.4 ± 0.2	41.5 ± 0.7	50.1 ± 1	17.3 ± 0.3	34.6 ± 0.4	1016 ± 76	3 ± 0
	Day 28	8.95 ± 0.5	5.79 ± 1.93	15.5 ± 0.8	44.9 ± 3.1	50.2 ± 1.1	17.3 ± 0.3	34.5 ± 0.7	999 ± 242	n.d.
	Day 90	8.50 ± 0.08	6.53 ± 2.43	14.5 ± 0.7	42.1 ± 1.5	49.5 ± 1.3	17.1 ± 0.6	34.5 ± 0.5	955 ± 71	1 ± 1
Sham	Day 2	8.35 ± 0.10	12.23 ± 1.99	14.8 ± 0.3	42.8 ± 0.5	51.3 ± 0.2	17.7 ± 0.2	34.6 ± 0.4	917 ± 187	2 ± 0
	Day 10	8.30 ± 0.10	9.23 ± 1.40	14.5 ± 0.7	42.5 ± 1.7	51.2 ± 1.5	17.4 ± 0.7	34.0 ± 0.5	724 ± 197	3 ± 2
	Day 28	8.42 ± 0.43	5.96 ± 0.55	14.9 ± 0.6	44.1 ± 1.9	52.4 ± 0.9	17.7 ± 0.3	33.8 ± 0.4	997 ± 31	n.d
	Day 90	8.47 ± 1.18	7.61 ± 3.79	13.9 ± 3.4	39.9 ± 9.5	46.5 ± 6.1	16.2 ± 2.3	34.8 ± 0.6	1128 ± 69	4 ± 2

Table S8. Haematological parameters in female rats. Ordered by experimental group, the descriptive statistics of centralization and dispersion (mean and standard deviation) and the statistical significance obtained by the one-way non-parametric ANOVA (Kruskal-Wallis test, according to the levels of significance: (*) p <0.05 and (**) p <0.01 are included). MI-ADSC-CS, MI-CS and Sham-ADSC-CS vs. Sham control group. Analysis was performed in 3 animals per each experimental group and sex for day 2 and 10 post-implant and in 5 animals for day 28 and 90 post-implant. Reticulocytes were not determined (n.d.) on Day 28.

FEMALE	Time of analysis	RBC (×10^{6}cell/ml)	WBC (×10^{3}cell/ml)	Haemoglobin (g/dl)	Hematocrit (%)	MCV (fL)	MCH (pg)	MCHC (g/dl)	Platelet (×10^{3}cell /ml)	Reticulocytes (%)
MI-ADSC-CS	Day 2	7.11 ± 0.32	3.90 ± 1.18	13.1 ± 0.7	38.7 ± 1.4	54.5 ± 0.4	18.4 ± 0.3	33.7 ± 0.7	665 ± 35	3 ± 0
	Day 10	7.75 ± 0.12	3.59 ± 1.22	14.2 ± 0.1	41.3 ± 0.8	53.3 ± 0.2	18.3 ± 0.4	34.3 ± 0.8	936 ± 15	2 ± 0
	Day 28	8.60 ± 0.43	4.01 ± 2.34	15.7 ± 0.3	45.6 ± 0.9	51.6 ± 0.7	17.8 ± 0.3	34.5 ± 0.2	1010 ± 85	n.d.
	Day 90	8.21 ± 0.39	3.57 ± 1.03	14.7 ± 0.3	43.1 ± 0.5	52.6 ± 2.1	17.9 ± 0.4	34.0 ± 0.5	921 ± 77	1 ± 1
MI-CS	Day 2	8.00 ± 0.30	3.97 ± 1.42	14.5 ± 0.3	42.6 ± 1.8	53.3 ± 0.4	18.2 ± 0.4	34.1 ± 0.8	864 ± 135	2 ± 1
	Day 10	7.84 ± 0.26	3.39 ± 0,67	14.3 ± 0,5	41.7 ± 1.2	53.2 ± 0.9	18.2 ± 0	34.2 ± 0.7	1255 ± 89	3 ± 1
	Day 28	9.34 ± 1.14	3.68 ± 2.32	16.8 ± 1.7	48.0 ± 3.9	51.6 ± 2.3	18.0 ± 0.5	35.0 ± 0.9	1101 ± 231	n.d.
	Day 90	8.98 ± 1.84	4.63 ± 0.77	16.1 ± 2.9	46.0 ± 8.1	51.4 ± 1.3	18.0 ± 0.4	35.0 ± 0.2	751 ± 105	1 ± 1
Sham-ADSC-CS	Day 2	7.47 ± 0.31	4.57 ± 0.20	13.4 ± 0.40	38.9 ± 1.8	52.2 ± 0.5	18.0 ± 0.3	34.4 ± 0.5	821 ± 61	2 ± 1
	Day 10	7.50 ± 0.48	3.87 ± 0.65	13.9 ± 0.8	40.0 ± 1.8	53.4 ± 1.1	18.5 ± 0.2	34.7 ± 0.4	1031 ± 80	2 ± 0
	Day 28	8.37 ± 0.13	4.39 ± 1.26	15.0 ± 0.1	43.9 ± 0.4	52.5 ± 0.6	18.0 ± 0.3	34.2 ± 0.2	835 ± 100	n.d.
	Day 90	8.86 ± 0.75	5.63 ± 1.91	15.6 ± 1.3	45.8 ± 3.7	51.7 ± 0.7	17.6 ± 0.4	34.1 ± 0.3	879 ± 128	2 ± 1
Sham	Day 2	7.19 ± 0.39	5.29 ± 1.37	13.2 ± 0.9	38.5 ± 1.8	53.6 ± 0.6	18.4 ± 0.3	34.2 ± 1	747 ± 62	2 ± 0
	Day 10	6.80 ± 0.18	2.80 ± 0.64	13.1 ± 0.2	39.2 ± 1.1	57.7 ± 3.3	19.2 ± 0.8	33.3 ± 0.4	944 ± 51	3 ± 1
	Day 28	8.51 ± 0.39	4.65 ± 0.68	15.2 ± 0.8	44.7 ± 2	52.5 ± 1.1	17.9 ± 0.2	34.1 ± 0.4	822 ± 90	n.d.
	Day 90	8.37 ± 0.27	3.69 ± 1.64	14.4 ± 0.6	41.6 ± 1.6	49.6 ± 0.6	17.2 ± 0.4	34.6 ± 0.3	887 ± 93	3 ± 1

Table S9. Urine Biochemistry parameters for males. Ordered by experimental group, the descriptive statistics of centralization and dispersion (mean and standard deviation) and the statistical significance obtained in the one-way non-parametric ANOVA (Kruskal-Wallis test, according to the levels of significance: (*) p <0.05 and (**) p <0.01 are included). MI-ADSC-CS, MI-CS and Sham-ADSC-CS vs. Sham control group results are represented. Analysis was performed in 3 animals per each experimental group and sex for day 2 and 10 post-implant and in 5 animals for day 90 post-implant. Nitrites are represented as a positive or negative (in brackets the number of animals positive or negative from the total animals).

MALE		Density (mg/dl)	pH	Leukocytes (cel/µl)	Nitrites	Proteins (mg/dl)	Glucose	Ketonic bodies (mg/dl)	Urobilinogen (mg/dl)	Bilirubine (mg/dl)	Erythrocytes (cel/µl)
**MI-ADSC-CS**	**Day 2**	1018 ± 3	6.3 ± 0.2	50 ± 43	Negative (3/3)	41 ± 0	Normal (5/5)	8.3 ± 5.7	Normal (3/3)	0.0 ± 0.0	20.0 ± 8.7
	**Day 10**	1010 ± 9	6.8 ± 0.2	0 ± 0	Negative (2/3)	42 ± 29	Normal (5/5)	6.6 ± 7.6	Normal (3/3)	0.3 ± 0.5	6.6 ± 5.7
	**Day 90**	1019 ± 9	6.4 ± 0.2	40 ± 0	Negative (3/5)	45 ± 29	Normal (5/5)	13.0 ± 7.6	Normal (5/5)	0.0 ± 0.0	9.1 ± 5.7
**MI-CS**	**Day 2**	1021 ± 3	6.3 ± 0.2	75 ± 43	Negative (2/3)	75 ± 0	Normal (5/5)	15.0 ± 0.0	Normal (3/3)	0.0 ± 0.0	7.3 ± 5.7
	**Day 10**	1007 ± 3	7.0 ± 0	13 ± 18	Negative (2/3)	25 ± 0	Normal (5/5)	0.0 ± 0.0	Normal (2/2)	0.0 ± 0.0	5.0 ± 7.0
	**Day 90**	1020 ± 5	6.2 ± 0.2	65 ± 49	Positive (3/5)	35 ± 22	Normal (5/5)	14.0 ± 21.0	Normal (5/5)	0.2 ± 0.4	11.1 ± 8.4
**Sham-ADSC-CS**	**Day 2**	1022 ± 3	6.0 ± 0	100 ± 0	Negative (2/3)	66 ± 72	Normal (5/5)	15.0 ± 0.0	Normal (3/3)	0.0 ± 0.0	100.6 ± 130.9
	**Day 10**	1014 ± 3	7.0 ± 0	17 ± 13	Positive (2/3)	25 ± 0	Normal (5/5)	1.6 ± 2.8	Normal (3/3)	0.0 ± 0.0	3.3 ± 5.7
	**Day 90**	1010 ± 8	6.5 ± 0.7	50 ± 70	Negative (5/5)	13 ± 17	Normal (5/5)	2.5 ± 3.5	Normal (5/5)	0.0 ± 0.0	5.3 ± 7.1
**Sham**	**Day 2**	1023 ± 3	6.2 ±0.2	25 ± 0	Negative (2/3)	42 ± 29	Normal (5/5)	12.0 ± 5.7	Normal (3/3)	0.7 ± 0.6	20.0 ± 8.6
	**Day 10**	1013 ± 3	7.3 ± 0.6	25 ± 0	Positive (2/3)	25 ± 0	Normal (5/5)	0.0 ± 0.0	Normal (3/3)	0.0 ± 0.0	0.0 ± 0.0
	**Day 90**	1018 ± 6	6.2 ± 0.0	100 ± 22	Negative (3/5)	35 ± 20	Normal (4/5)	15.0 ± 20.3	Normal (5/5)	0.0 ± 0.0	19.0 ± 8.2

Table S10. Urine Biochemistry parameters for males. Ordered by experimental group, the descriptive statistics of centralization and dispersion (mean and standard deviation) and the statistical significance obtained by the one-way non-parametric ANOVA (Kruskal-Wallis test, according to the levels of significance (*) *p* <0.05 and (**) *p* <0.01 are included). MI-ADSC-CS, MI-CS and Sham-ADSC-CS vs. Sham control group results are represented. Analysis was performed in 3 animals per each experimental group and sex for day 2 and 10 post-implant and in 5 animals for day 90 post-implant. Nitrites are represented as a positive or negative (in brackets the number of animals positive or negative from the total animals).

FEMALE		Density (mg/dl)	pH	Leukocytes (cel/µl)	Nitrites	Proteins (mg/dl)	Glucose	Ketonic bodies (mg/dl)	Urobilinogen (mg/dl)	Bilirubine (mg/dl)	Erythrocytes (cel/µl)
**MI-ADSC-CS**	**Day 2**	1017 ± 6	6.5 ± 0.5	16 ± 14	Negative (3/3)	16 ± 13	Normal (3/3)	2.1 ± 2.9	Normal (3/3)	0.6 ± 0.6	11.6 ± 12.5
	**Day 10**	1016 ± 10	6.5 ± 0.5	8 ± 14	Negative (3/3)	33 ± 38	Normal (3/3)	5.3 ± 8.6	Normal (3/3)	0.3 ± 0.6	0.0 ± 0.0
	**Day 90**	1012 ± 10	8.3 ± 0.5	183 ± 274	Positive (2/3)	176 ± 281	Normal (3/3)	5.3 ± 8.6	Normal (2/3)	0.0 ± 0.0	11.6 ± 12.5
**MI-CS**	**Day 2**	1017 ± 6	6.5 ± 0.5	16 ± 14	Negative (2/3)	33 ± 38	Normal (3/3)	5.3 ± 8.6	Normal (3/3)	0.6 ± 0.0	8.3 ± 4.4
	**Day 10**	1017 ± 6	7.0 ± 1.0	8 ± 14	Negative (2/3)	25 ± 0	Normal (3/3)	0.0 ± 0.0	Normal (3/3)	0.0 ± 0.0	20.0 ± 26.4
	**Day 90**	1012 ± 8	7.5 ± 1.0	8 ± 14	Positive (3/3)	8 ± 14	Normal (3/3)	0.0 ± 0.0	Normal (3/3)	0.0 ± 0.0	0 ± 0
**Sham-ADSC-CS**	**Day 2**	1021 ± 9	6.3 ± 0.5	66 ± 57	Negative (2/3)	33 ± 38	Normal (3/3)	10.6 ± 8.6	Normal (2/3)	0.0 ± 0.0	25 ± 25.0
	**Day 10**	1018 ± 3	6.5 ± 0.0	16 ± 14	Negative (2/3)	33 ± 38	Normal (3/3)	3.3 ± 2.9	Normal (2/3)	0.0 ± 0.0	11.6 ± 12.5
	**Day 90**	1017 ± 6	6.2 ± 0.7	20 ± 11	Negative (5/5)	20 ± 11	Normal (5/5)	1.1 ± 2.23	Normal (5/5)	0.2 ± 0.4	7.0 ± 10.9
**Sham**	**Day 2**	1025 ± 0	5.6 ± 0.5	25 ± 0	Positive (2/3)	58 ± 28	Normal (3/3)	23.3 ± 23.6	Normal (2/3)	1.0 ± 0.0	15.0 ± 8.6
	**Day 10**	1021 ± 7	6.0 ± 0.9	16 ± 14	Positive (2/3)	33 ± 38	Normal (3/3)	6.6 ± 7.6	Normal (2/3)	0.0 ± 0.0	8.3 ± 14.4
	**Day 90**	1019 ± 4	6.2 ± 0.2	143 ± 237.	Negative (4/5)	20 ± 11	Normal (5/5)	6.8 ± 5.47	Normal (5/5)	0.0 ± 0.0	6.0 ± 5.5

Table S11. Relative weight of organs in males. The weight of each organ was normalized against the net weight of the animals. The data obtained from the animals euthanized at days 2, 10 and 90 is shown. Ordered by experimental group, the descriptive statistics of centralization and dispersion (mean and standard deviation) and the statistical significance obtained by the one-way non-parametric ANOVA (Kruskal-Wallis test, according to the levels of significance (*) p <0.05 and (**) p <0.01 are included). MI-ADSC-CS, MI-CS and Sham-ADSC-CS vs Sham control group results are represented. Analysis was performed in 3 animals per each experimental group and sex for day 2 and 10 post-implant and in 5 animals for day 90 post-implant.

MALES		Relative weight of organs (%)										
		Spleen	Brain	Heart	Liver	Thyroids	Kidney		Adrenals		Testis	
							Right	Left	Right	Left	Right	Left
MI-ADSC-CS	Day 2	0.200 ± 0.023	0.561 ± 0.036	0.251 ± 0.139	3.651 ± 0.243	0.009 ± 0.002	0.393 ± 0.020	0.366 ± 0.022	0.011 ± 0.001	0.011 ± 0.001	0.376 ± 0.070	0.416 ± 0.056
	Day 10	0.198 ± 0.031	0.578 ± 0.030	0.366 ± 0.021	3.015 ± 0.235	0.021 ± 0.008	0.344 ± 0.014	0.347 ± 0.022	0.010 ± 0.002	0.012 ± 0.001	0.425 ± 0.011	0.499 ± 0.176
	Day 90	0.185 ± 0.033	0.508 ± 0.086	0.354 ± 0.087	3.288 ± 0.148	0.023 ± 0.005	0.313 ± 0.030	0.313 ± 0.034	0.009 ± 0.002	0.010 ± 0.001	0.312 ± 0.038	0.365 ± 0.058
MI-CS	Day 2	0.193 ± 0.006	0.533 ± 0.048	0.394 ± 0.041	3.634 ± 0.174	0.028 ± 0.008	0.361 ± 0.024	0.361 ± 0.004	0.010 ± 0.003	0.011 ± 0.001	0.410 ± 0.039	0.404 ± 0.034
	Day 10	0.175 ± 0.009	0.576 ± 0.058	0.341 ± 0.020	3.129 ± 0.174	0.032 ± 0.004	0.358 ± 0.009	0.341 ± 0.011	0.011 ± 0.001	0.013 ± 0.003	0.458 ± 0.036	0.459 ± 0.032
	Day 90	0.146 ± 0.014	0.454 ± 0.021	0.285 ± 0.022	3.059 ± 0.220	0.020 ± 0.004	0.310 ± 0.019	0.310 ± 0.015	0.009 ± 0.003	0.008 ± 0.001	0.341 ± 0.021	0.333 ± 0.025
Sham-ADSC-CS	Day 2	0.198 ± 0.020	0.555 ± 0.022	0.310 ± 0.014	3.882 ± 0.205	0.029 ± 0.003	0.392 ± 0.021	0.388 ± 0.009	0.011 ± 0.002	0.010 ± 0.002	0.414 ± 0.024	0.443 ± 0.028
	Day 10	0.207 ± 0.006	0.521 ± 0.014	0.352 ± 0.014	3.448 ± 0.100	0.037 ± 0.001	0.356 ± 0.022	0.370 ± 0.019	0.012 ± 0.001	0.011 ± 0.002	0.419 ± 0.022	0.409 ± 0.018
	Day 90	0.142 ± 0.003	0.440 ± 0.003	0.333 ± 0.010	3.170 ± 0.035	0.137 ± 0.163	0.323 ± 0.004	0.331 ± 0.022	0.007 ± 0.000	0.006 ± 0.001	0.345 ± 0.002	0.353 ± 0.003
Sham	Day 2	0.197 ± 0.012	0.538 ± 0.031	0.280 ± 0.019	3.723 ± 0.369	0.008 ± 0.001	0.370 ± 0.010	0.381 ±0.007	0.010 ± 0.002	0.010 ± 0.002	0.420 ± 0.040	0.410 ± 0.048
	Day 10	0.185 ± 0.015	0.601 ± 0.022	0.292 ± 0.011	3.223 ± 0.240	0.031 ± 0.005	0.375 ± 0.029	0.367 ± 0.025	0.010 ± 0.002	0.011 ± 0.002	0.456 ± 0.024	0.453 ± 0.018
	Day 90	0.155 ± 0.014	0.461 ± 0.039	0.311 ± 0.026	3.507 ± 0.308	0.020 ± 0.004	0.338 ± 0.022	0.334 ± 0.031	0.010 ± 0.002	0.007 ± 0.001	0.359 ± 0.041	0.359 ± 0.045

Table S12. Relative weight of organs in females. The weight of each organ was normalized against the net weight of the animals. The data obtained from the animals euthanized at days 2, 10 and 90 is shown. Ordered by experimental group, the descriptive statistics of centralization and dispersion (mean and standard deviation) and the statistical significance obtained by the one-way non-parametric ANOVA (Kruskal-Wallis test, according to the levels of significance (*) p <0.05 and (**) p <0.01 are included). MI-ADSC-CS, MI-CS and Sham-ADSC-CS vs Sham control group results are represented. Analysis was performed in 3 animals per each experimental group and sex for day 2 and 10 post-implant and in 5 animals for day 90 post-implant.

**FEMALES**		**Relative weight of organs (%)**										
		**Spleen**	**Brain**	**Heart**	**Liver**	**Thyroids**	**Kidney**		**Adrenals**		**Ovary**	
							**Right**	**Left**	**Right**	**Left**	**Right**	**Left**
**MI-ADSC-CS**	**Day 2**	0.199 ± 0.015	0.926 ± 0.050	0.428 ± 0.065	3.681 ± 0.252	0.011 ± 0.003	0.382 ± 0.023	0.389 ± 0.029	0.022 ± 0.003	0.021 ± 0.004	0.028 ± 0.007	0.027 ± 0.006
	**Day 10**	0.230 ± 0.027	0.954 ± 0.025	0.393 ± 0.031	3.252 ± 0.382	0.024 ± 0.001	0.247 ± 0.214	0.248 ± 0.215	0.020 ± 0.001	0.022 ± 0.003	0.026 ± 0.005	0.026 ± 0.005
	**Day 90**	0.162 ± 0.049	0.803 ± 0.009	0.397 ± 0.023(*)	3.136 ± 0.130	0.012 ± 0.002(*)	0.359 ± 0.014	0.349 ± 0.009	0.016 ± 0.002	0.019 ± 0.002	0.019 ± 0.005	0.023 ± 0.003
**MI-CS**	**Day 2**	0.213 ± 0.012	0.906 ± 0.076	0.435 ± 0.045	3.758 ± 0.224	0.040 ± 0.011	0.3983 ± 0.013	0.399 ± 0.021	0.020 ± 0.001	0.020 ± 0.001	0.024 ± 0.006	0.024 ± 0.004
	**Day 10**	0.198 ± 0.027	0.890 ± 0.046	0.455 ± 0.037	3.410 ± 0.396	0.041 ± 0.004	0.370 ± 0.010	0.383 ± 0.020	0.016 ± 0.003	0.018 ± 0.004	0.022 ± 0.004	0.026 ± 0.005
	**Day 90**	0.185 ± 0.021	0.813 ± 0.134	0.383 ± 0.065	3.370 ± 0.394	0.011 ± 0.003 (*)	0.349 ± 0.010	0.359 ± 0.011	0.015 ± 0.003	0.017 ± 0.002	0.018 ± 0.001	0.018 ± 0.003
**Sham-ADSC-CS**	**Day 2**	0.229 ± 0.004	0.877 ± 0.076	0.339 ± 0.006	4.233 ± 0.394	0.038 ± 0.012	0.402 ± 0.020	0.406 ± 0.020	0.024 ± 0.001	0.024 ± 0.001	0.020 ± 0.003	0.024 ± 0.006
	**Day 10**	0.210 ± 0.014	0.944 ± 0.038	0.419 ± 0.085	3.307 ± 0.054	0.048 ± 0.001	0.376 ± 0.012	0.377 ± 0.015	0.020 ± 0.001	0.022 ± 0.001	0.025 ± 0.001	0.026 ± 0.001
	**Day 90**	0.208 ± 0.019(*)	0.833 ± 0.076	0.355 ± 0.034	3.219 ± 0.412	0.039 ± 0.007	0.370 ± 0.016	0.373 ± 0.026	0.020 ± 0.187	0.019 ± 0.004	0.029 ± 0.002(*)	0.036 ± 0.006(*)
**Sham**	**Day 2**	0.216 ± 0.014	0.879 ± 0.072	0.357 ± 0.031	3.833 ± 0.150	0.009 ± 0.003	0.405 ± 0.025	0.387 ± 0.008	0.020 ± 0.001	0.021 ± 0.001	0.023 ± 0.003	0.026 ± 0.002
	**Day 10**	0.228 ± 0.019	0.886 ± 0.019	0.348 ± 0.021	3.427 ± 0.183	0.040 ± 0.012	0.372 ± 0.014	0.3763 ± 0.019	0.019 ± 0.001	0.021 ± 0.003	0.021 ± 0.002	0.025 ± 0.005
	**Day 90**	0.157 ± 0.009	0.757 ± 0.031	0.305 ± 0.025	3.189 ± 0.331	0.036 ± 0.005	0.358 ± 0.033	0.358 ± 0.030	0.017 ± 0.002	0.017 ± 0.001	0.019 ± 0.004	0.019 ± 0.002
